# Trends in the application of remote sensing in blue carbon science

**DOI:** 10.1002/ece3.10559

**Published:** 2023-09-23

**Authors:** Rocio Araya‐Lopez, Micheli Duarte de Paula Costa, Melissa Wartman, Peter I. Macreadie

**Affiliations:** ^1^ Centre for Integrative Ecology, School of Life and Environmental Sciences Deakin University Burwood Victoria Australia

**Keywords:** bibliometrics, blue carbon ecosystem, mangrove, nature‐based solution, saltmarsh, seagrass

## Abstract

Blue carbon ecosystems (BCEs), such as mangroves, saltmarshes, and seagrasses, are increasingly recognized as natural climate solutions. Evaluating the current extent, losses, and gains of BCEs is crucial to estimating greenhouse gas emissions and supporting policymaking. Remote sensing approaches are uniquely suited to assess the factors driving BCEs dynamics and their impacts at various spatial and temporal scales. Here, we explored trends in the application of remote sensing in blue carbon science. We used bibliometric analysis to assess 2193 published papers for changes in research focus over time (1990 – June 2022). Over the past three decades, publications have steadily increased, with an annual growth rate of 16.9%. Most publications focused on mangrove ecosystems and used the optical spaceborne Landsat mission, presumably due to its long‐term, open‐access archives. Recent technologies such as LiDAR, UAVs, and acoustic sensors have enabled fine‐scale mapping and monitoring of BCEs. Dominant research topics were related to mapping and monitoring natural and human impacts on BCEs, estimating vegetation and biophysical parameters, machine and deep learning algorithms, management (including conservation and restoration), and climate research. Based on corresponding author affiliations, 80 countries contributed to the field, with United States (27.2%), China (15.0%), Australia (7.5%), and India (6.0%) holding leading positions. Overall, our results reveal the need to increase research efforts for seagrasses, saltmarshes, and macroalgae, integrate technologies, increase the use of remote sensing to support carbon accounting methodologies and crediting schemes, and strengthen collaboration and resource sharing among countries. Rapid advances in remote sensing technology and decreased image acquisition and processing costs will likely enhance research and management efforts focused on BCEs.

## Graphical abstract

Our findings highlight the various applications of remote sensing technologies to further our understanding of blue carbon ecosystems and their carbon stocks, greenhouse gas fluxes, changes in extent through space and time, and support ecosystem management, including restoration and conservation efforts.

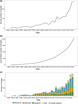

## 1 INTRODUCTION

In recent years, natural climate solutions have become an emerging option to mitigate and adapt to climate change impacts (Griscom et al., 2017). Natural climate solutions focus on ecosystem management approaches (e.g., conservation, restoration, and reforestation) to enhance carbon sequestration and reduce greenhouse gas emissions. Blue carbon ecosystems (BCEs), such as mangroves, saltmarshes, and seagrasses, are one of nature's most efficient, long‐term natural climate solutions (Macreadie et al., 2021). These ecosystems have exceptional carbon sequestration and storage capacity and provide considerable co‐benefits, including water filtration, fishery production, pollution buffering, and coastline protection (Friess et al., 2020; Himes‐Cornell et al., 2018).

However, BCEs rank among the most threatened ecosystems worldwide and continue to decline (Bunting et al., 2022; Murray et al., 2022; Turschwell et al., 2021; Waycott et al., 2009), with the main drivers of degradation being land‐use change and climate change impacts, including sea‐level rise (Lovelock & Reef, 2020; Pendleton et al., 2012). When degraded or destroyed, BCEs become a significant source of greenhouse gases, contributing further to climate change (Lovelock et al., 2017; Pendleton et al., 2012). The restoration and conservation of BCEs can therefore play a significant role in mitigating and adapting to climate change while providing additional co‐benefits for communities (Macreadie et al., 2021).

Although a focus on natural climate solutions is providing significant impetus for restoring and conserving BCEs, such approaches are limited by a lack of understanding of the past and current spatial distribution of BCE habitats (Lovelock & Reef, 2020; Macreadie et al., 2019). For instance, the extent of BCE losses or expansion due to climate change, as well as temporal changes in ecosystem structure and ecological process associated with carbon cycling, are often poorly known (Lovelock & Reef, 2020; Macreadie et al., 2019).

Remote sensing (RS) provides cost‐effective tools for mapping, monitoring, and estimating biophysical parameters for terrestrial and marine ecosystems (Pham et al., 2019; Turner et al., 2003). RS technologies measure the energy reflected or emitted by the Earth's surface at different wavelengths, through sensors mounted on satellites, aerial vehicles, and ground‐based instruments (Lechner et al., 2020). Satellite‐based multispectral sensors have been broadly available since 1972 (e.g., Landsat missions; Powell et al., 2007). Significant advances in spatial resolution by commercial satellite‐based sensors (e.g., Worldview, QuickBird, Planet) have provided new opportunities for habitat mapping at finer spatial scales (e.g., pixel size <10 m^2^; Nagendra et al. 2013), while very high spatial resolution images from uncrewed aerial systems or vehicles (UAS/UAVs) have provided unprecedented spatial resolutions unmatched by satellite imagery (Ventura et al., 2018). In tandem with increased spatial resolution, hyperspectral sensors have offered new opportunities for plant species classification, biophysical properties estimation, soil properties monitoring, and habitat mapping (Schmidt & a. K. Skidmore., 2003; Ustin et al., 2004). As opposed to optical imagery, active sensors, such as synthetic aperture radar (SAR), can penetrate through vegetation canopy and are unaffected by clouds and haze, while light detection and ranging (LiDAR) provides ground elevation and vegetation structure data (Li et al., 2014). These sensors are uniquely suited to detect standing water, monitor soil moisture, estimate above‐ground biomass, and produce topographic maps. Other active sensors include acoustic techniques such as side‐scan sonar, and single‐ and multi‐beam echo sounders, which are commonly used approaches for mapping and monitoring submerged aquatic vegetation (Gumusay et al., 2019; Lecours et al., 2022).

The progressive development of sensors and platforms has increased the amount and complexity of RS data available for terrestrial and marine observations, leading to rapid and continuous advances in techniques and algorithms to process and retrieve more information from the data (Pettorelli et al., 2014). As RS products become more affordable and accessible, RS is becoming an important tool for biodiversity, conservation, and climate change research (Byrd et al., 2018; Lecours et al., 2022; Turner et al., 2003). Currently, blue carbon science is still a relatively new research field, and progressive developments in RS technologies offer opportunities for delivering the full potential of BCEs as natural climate solutions.

As a baseline to characterize this relatively new research field, this study provides a comprehensive assessment of the trends and developments of RS applications in blue carbon science through a bibliometric analysis. Our results provide valuable information to document the development of the field, identify research priorities, highlight advances in new technologies, and anticipate future research trends. We expect that the utilization of RS applications in blue carbon science is increasing over time, although the application may differ across ecosystem types. While freely available mid‐resolution imagery is the primary source to map and monitor blue carbon ecosystems, we predict changes in research focus over time.

## 2 METHODS

### 2.1 Data collection and extraction

The literature search included studies published in the primary literature from 1900 to June 2022, identified using Web of Science™ (Clarivate™; webofknowledge.com). Our search included titles, abstracts, keywords, and keyword plus (i.e., words or phrases that frequently appear in the titles of an article's references, but do not appear in the title of the article itself) of the Science Citation Index Expanded, the Social Science Citation Index, and the Emerging TSource Citation Index databases. A full list of BCE‐ and RS‐related query terms is available in Table S1. Only English‐language publications were considered in this analysis, and we acknowledge that our results are biased by the exclusion of non‐English‐language studies (Amano et al., 2021; Christie et al., 2021).

**FIGURE 1:**
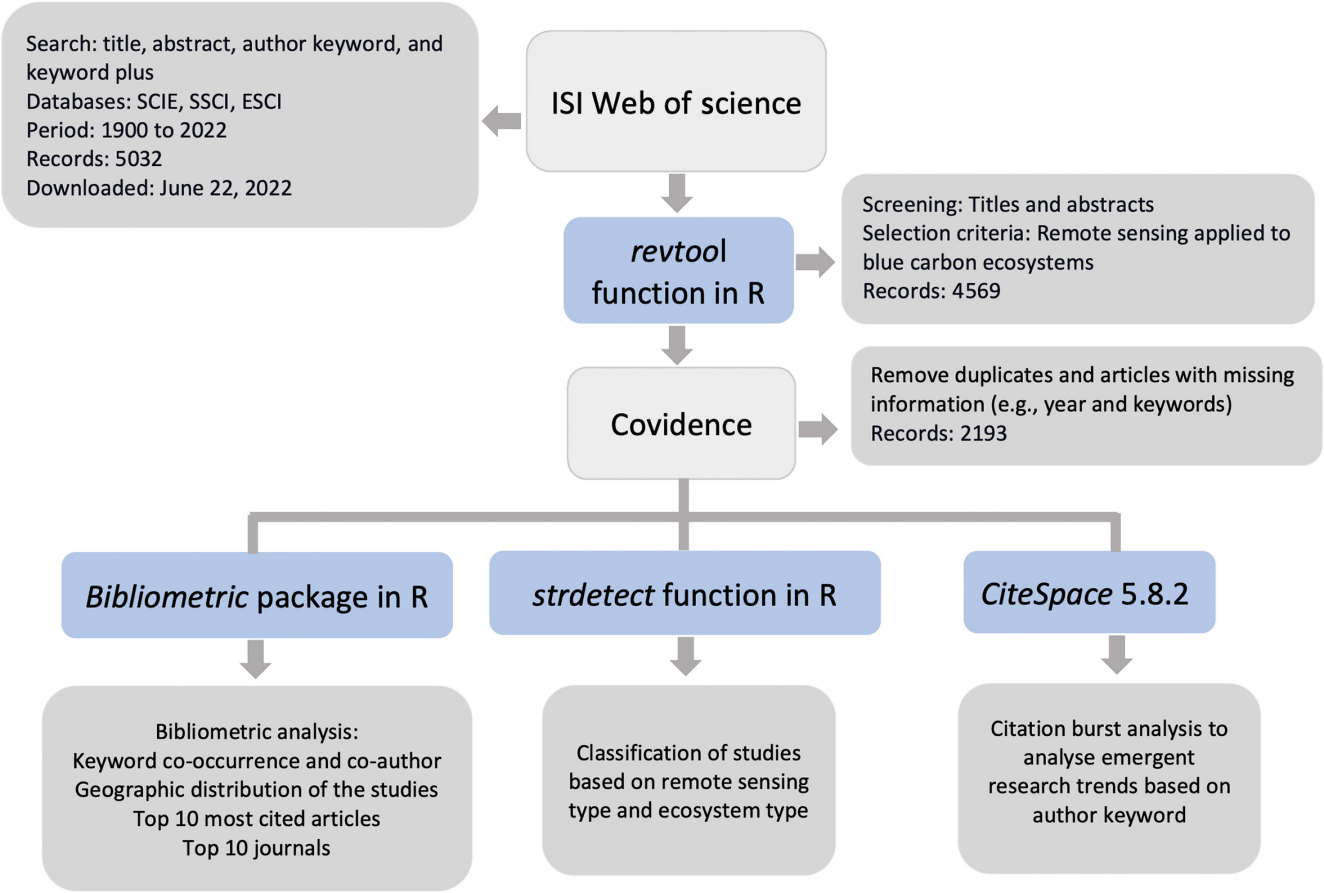
Diagram describing the methodology followed for the bibliometric analysis of remote sensing applications in blue carbon science (1900–2022).

The dataset was downloaded on 22 June 2022, and the original search retrieved 5032 bibliographic records. The dataset was screened to double‐check the search criteria and remove records with duplicate references and missing information (i.e., publication year and abstract) using the *revtool* package (Westgate, 2019) in R studio (RStudio Team, 2022). We manually examined titles and abstracts from the 4569 remaining records using the web‐based systematic review software Covidence (www.covidence.org) to select relevant publications using screening criteria (Table S2). We retained publications that focused, at least partially, on one or several BCEs and where RS was the primary approach to address the study's aims. We excluded papers that focused on non‐vegetated coastal environments (e.g., mudflats), coral reefs, microalgae blooms, and seaweed farming. Transitions are an important aspect of mangrove and saltmarshes processes covered by remote sensing applications. Our criteria excluded publications covering exclusively mudflat environments without considering blue carbon ecosystems (i.e., mangroves, saltmarshes, seagrasses, and macroalgae) and coastal wetlands. The screening process resulted in the identification of 2193 relevant publications (DOI: 10.5061/dryad.m0cfxpp8g). The resulting records were classified based on titles, abstracts, keywords, and keywords plus information, and according to RS type, BCE type, methodological approach, ecosystem variables measured, as well as main research objective. The full review methodology is presented in Figure 1.

### 2.2 Data analysis

To identify general patterns, we derived statistical summaries of bibliographic records and extracted information from each publication using the *bibliometrix* R package (Aria & Cuccurullo, 2017). From this analysis, we obtained the overall annual publication rate grouped by habitat and sensor type (1990–2022) and the most productive countries based on the first affiliation of the corresponding author. We used the *tidyverse* R package (Wickham et al., 2019) to visualize the geographic distribution of publications. We also conducted a co‐occurrence analysis of main keywords using the *bibliometrix* R package (Aria & Cuccurullo, 2017). Before the analysis, we combined similar keywords, such as “mangroves” and “mangrove” which were incorporated into the analysis as “mangrove.” To identify research directions, we used CiteSpace version 5.8.2 (Chen, 2006) to perform a citation burst history analysis based on keywords. Lastly, we used the *strdetect* function in R studio (RStudio Team, 2022) to search studies containing specific keywords related to the main RS technologies (e.g., Landsat, MODIS, UAV, LiDAR) and their applications (e.g., mapping, monitoring) to blue carbon science (Table S9).

## 3 RESULTS & DISCUSSION

**FIGURE 2:**
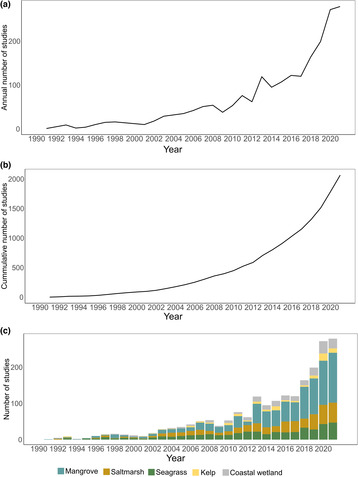
Temporal trends in publications focusing on applying remote sensing to blue carbon ecosystems, based on a total of 2193 publications published from 1990 to 2021. (a) Distribution of the annual number of publications, (b) Cumulative number of publications, and (c) Number of publications on single habitats, multiple habitats, and coastal wetlands in general (i.e., studies that did not specify habitat types). For visualization purposes, the studies from 2022 were not included in the graph, as they only encompassed studies conducted until June of that year.

The bibliometric database compiled 2193 records for a 30‐year study period (Figure 2b). According to the classification of the WOS database, it comprises 2085 articles, 64 proceedings articles, 35 reviews, and 9 publications of other types (i.e., notes, editorial material, letters). Of this total, 43% focused on mangroves, 21% on saltmarshes, 20% on seagrasses, 5% on macroalgae (kelp), and 11% on coastal wetlands (i.e., studies that did not specify habitat types) in general (Figure 2c).

### 3.1 Publication trends

The average annual publication growth rate was 16.9% (Figure 2). The number of publications increased rapidly after 2001, although there were temporal fluctuations between 2009 and 2014, marked by alternating periods of growth and decline in publication trends. An upward trend was observed after 2017, with a notable increase in publications after 2020. We suggest that the growth in the number of publications in the mid‐2000s might relate to three main events: (1) the 2008 free access policy for Landsat records, which offered additional opportunities to monitor the Earth's surface (Turner et al., 2015), (2) the introduction, in 2009, of the term “blue carbon” to highlight the role of BCEs in mitigating climate change through carbon sequestration (Nellemann et al., 2009), and (3) to the increasing recognition of natural climate solutions in national climate goals (also known as Nationally Determined Contributions, NDCs) under the Paris Agreement in 2015 (Gallo et al., 2017).

Through our analysis, we found that the 2193 publications referencing RS were published across 360 journals (Table S3). The top 10 most productive journals published 40% of the total number of publications (*n* = 885 publications; Figure S1) and were spread across journals focused on RS science and applications, as well as journals focusing on wetland and coastal studies. The most productive journal was *Remote Sensing* (IF = 5.349), followed by *Estuarine Coastal and Shelf Science* (IF = 3.229), *International Journal of Remote Sensing* (IF = **3.531**), *Journal of Coastal Research* (IF = 1.110), and *Remote Sensing of Environment*, each publishing between 11.9% and 4.2% of the total number of publications (Table S3). The journals with the highest impact factors, including *Science* (IF = 63.832), *Nature Climate Change* (IF = 28.862), *Nature Sustainability* (IF = 27.157), and *Nature Communications* (IF = 17.694), only published one article each between 1900 and 2022 (Table S4).

We identified the most influential articles on remote sensing applications to BCEs based on their citation numbers (Table S5). The most highly cited paper (*n* = 3075 citations) was Giri et al. (2011) published in *Global Ecology and Biogeography*. Giri et al. (2011) used Landsat imagery from 2000 to deliver the highest resolution (30 m) and most comprehensive global mangrove distribution extent map at the time. This seminal publication and the associated global mangrove distribution map were used in several other studies of mangroves as natural climate solutions and potential carbon sinks (Hamilton & Casey, 2016; Hamilton & Friess, 2018; Sanderman et al., 2018; Simard et al., 2019). The second most cited paper (*n* = 461 citations), also published in *Global Ecology and Biogeography*, introduced the Bio‐ORACLE project, a global satellite‐based dataset for modeling shallow marine species distribution (Tyberghein et al., 2012). This ready‐to‐use marine environmental dataset provides high‐resolution ecological variables controlling the growth, survival, and distribution of BCEs (e.g., temperature and salinity). The increased accessibility of marine environmental data allowed an emerging body of literature aiming to predict climate‐induced species range shifts across space and time (Hu et al., 2021; Mchenry et al., 2021; van der Stocken et al., 2022).

The remainder of the 10 most cited papers (Table S5), most published before the 2000s, covered RS applications focused on mangrove ecosystems, using primarily optical imagery. In contrast, the 10 most cited publications in the last 5 years (Table S6) focused on habitat losses induced by human and climate change pressures at regional (Arias‐Ortiz et al., 2018) and global scales (Goldberg et al., 2020) by using time series analysis of freely available, mid‐resolution satellite imagery, and very‐high‐resolution data for species discrimination (Cao et al., 2018; Duffy et al., 2018; Ventura et al., 2018). We suggest that the increasing development of RS technologies, such as UAVs have offered the spatial resolution needed for accurately discriminating at the species level.

### 3.2 Global distribution of publications

**FIGURE 3:**
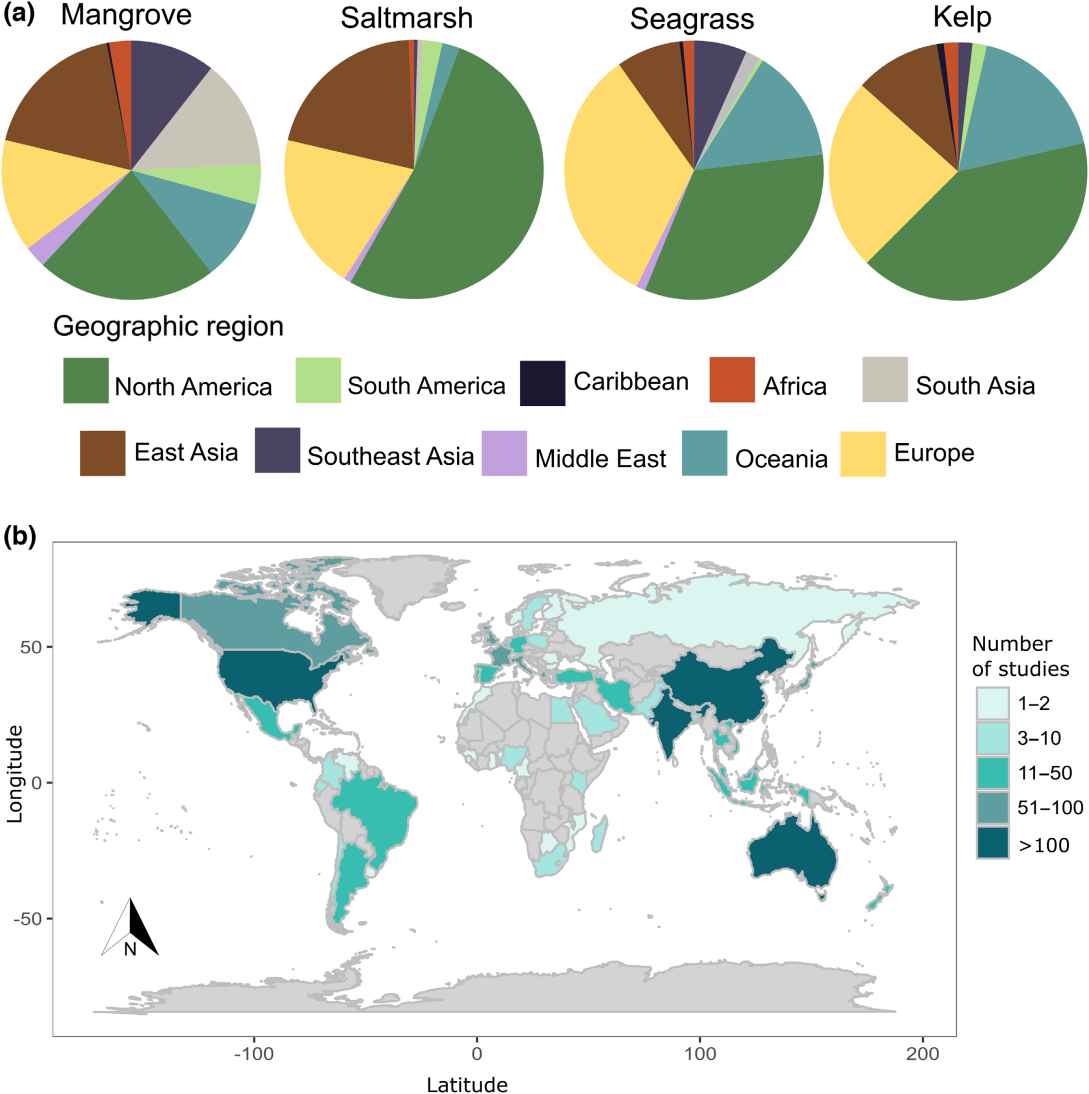
Geographic distribution of remote sensing publications applied to blue carbon ecosystems based on first‐author affiliations per habitat type (a) and total number of publications per country (b). The list of countries by geographic regions is shown in Table S7.

The contribution of different countries to the field was evaluated using the affiliation of the corresponding author of each publication (Figure 3b). From 1990 to 2022, 2181 authors from 80 countries, as represented by the corresponding author's affiliation, contributed to RS publications on BCEs. About 27.2% of the authors were affiliated with USA institutions, followed by China (15.0%), Australia (7.5%), and India (6.0%). European countries accounted for 20% of authors (mainly from the United Kingdom, France, Italy, Germany, and Spain). In comparison, American countries contributed around 9% (e.g., Canada, Brazil, Mexico, Argentina), and Southeast Asian countries contributed around 6% (e.g., Indonesia, Vietnam, Malaysia, Thailand, and Singapore) to the total number of publications. In contrast, our analysis found a lower contribution to the field by corresponding authors from African, Caribbean, and Middle Eastern countries. While our analysis provides some insight into the understanding of RS applications on a per‐country basis, our results are limited as the study areas included in the publications are not always aligned with the corresponding author's affiliation country. Furthermore, we only considered manuscripts published in English, which can underestimate our results. The observed variation in research contribution among countries can be attributed to factors such as research and development costs (such as hardware and software expenses, data processing and analysis costs, field survey expenditures), diverse expertise and capabilities of professionals in different regions, and available technological resources.

Additionally, we evaluated the contribution of countries to research by ecosystem type using the affiliation of the corresponding author of each publication (Figure 3a). We found that 57 countries contributed to mangrove research, with the USA, China, India, and Australia being the leading countries publishing this research for mangrove ecosystems. Seagrass studies were conducted by 47 countries, and kelp research was conducted by 23 countries, with the USA and Australia leading publications for both ecosystems. Saltmarsh research was contributed to by 28 countries with the USA and China leading the publications. A review of RS applications in BCEs by study location (*n* = 120) from 2010 to 2018 by Pham et al. (2019) found that mangrove research studies were conducted worldwide, while seagrass study sites were primarily located in South Asia and Europe, and saltmarsh studies were mainly conducted in the USA, Canada, and Europe. Our evaluation provides valuable insights into the global distribution of research efforts in BCEs. While the study has limitations, it highlights the need for greater collaboration and resource‐sharing among countries, especially for those holding large extensions of BCEs and threatened by anthropogenic and natural pressures, to enhance research and development in these critical ecosystems. As we move forward, continued efforts to expand our understanding of RS applications in BCEs will be essential to inform effective management and conservation strategies for these vital ecosystems.

### 3.3 Publication trends per habitat type

**FIGURE 4:**
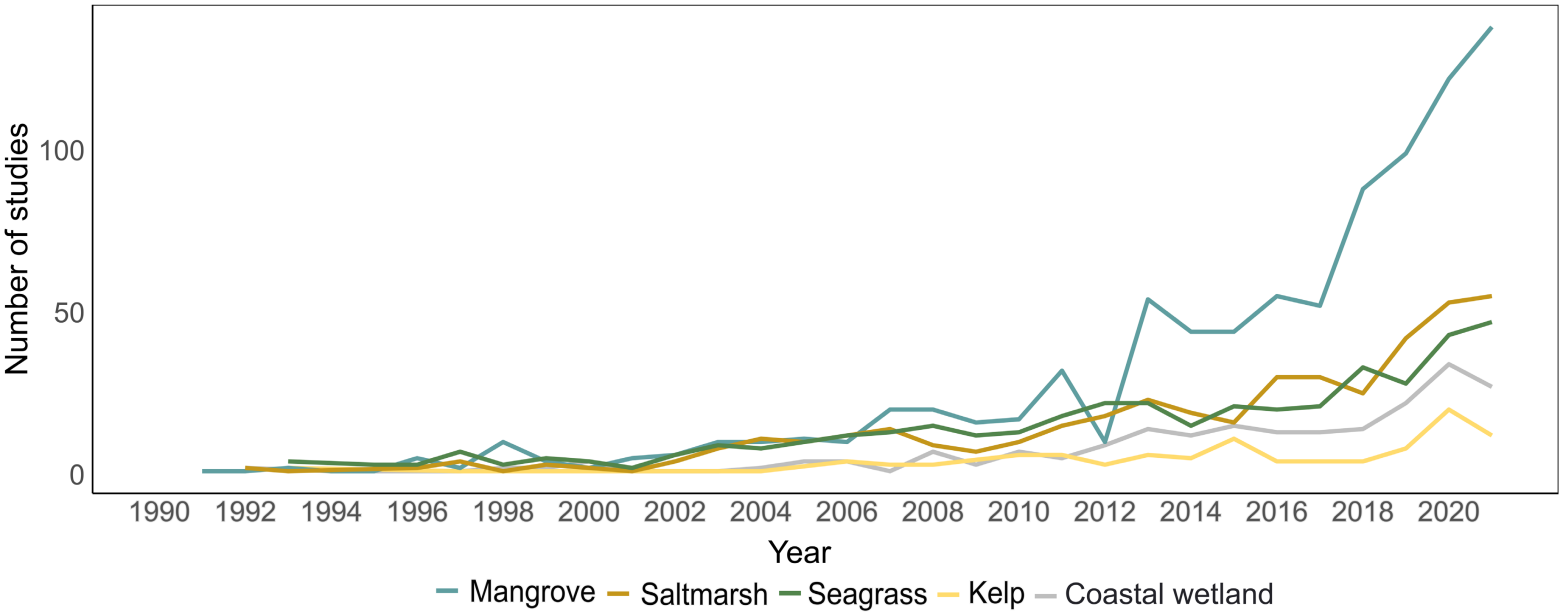
Number of publications applying remote sensing to single habitats and coastal wetlands (i.e., studies that did not specify habitat types) based on a total of 2193 publications published from 1990 to 2021. For visualization purposes, the studies from 2022 were not included in the graph, as they only encompassed studies conducted until June of that year.

The cumulative number of studies by habitat type has steadily increased over the 30‐year study period, with annual fluctuations (Figure 4). From 2009, the number of studies for all habitats has increased which coincided with the year when the term “blue carbon” was coined (Nellemann et al., 2009). We observed a significant divergence among BCEs studies applying remote sensing commencing in 2012, with considerable increase in research focused on mangroves. The greater number of publications focused on mangrove ecosystems can be attributed to (1) the increased international focus of mangrove management to mitigate and adapt to climate change (e.g., mangrove restoration, conservation and management plans are included in 45 NDCs) (Gallo et al., 2017), (2) their detectability through freely available, mid‐resolution satellite imagery due to their size and distribution within intertidal zones, in contrast to subtidal ecosystems like seagrasses and kelp, and (3) the rapid advances of Google Earth Engine in granting access to vast amounts of global and regional remote imagery, along with processing power, which could have contributed to these variations (Gorelick et al., 2017). Indeed, mangroves are often more studied not just because of their size, as kelp forest are also extensive. However, mapping subtidal seagrasses and kelp is hindered by many factors, including water reflection, scattering, and light absorption, limiting the application of RS technologies. RS applications can be particularly beneficial for countries with large mangrove cover, where anthropogenic and environmental issues are the most pressing, such as Indonesia, Myanmar, Malaysia, Philippines, Thailand, and Vietnam (Goldberg et al., 2020; Lovelock et al., 2015). Future remote sensing research should prioritize the development of accurate and effective methods for monitoring seagrass and saltmarsh ecosystems, given their critical ecological and socioeconomic value.

### 3.4 Remote sensing application to blue carbon ecosystems

**FIGURE 5:**
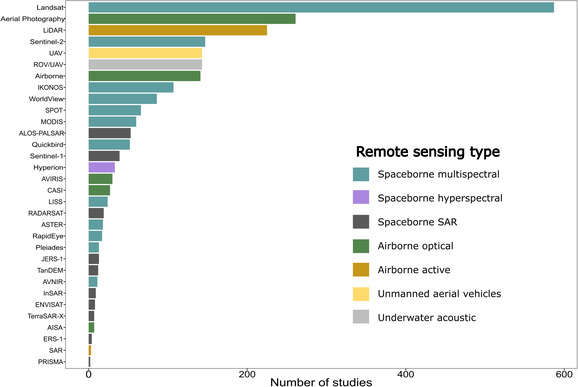
Number of published remote sensing studies in blue carbon science per sensor type, based on 1596 publications that mentioned sensor and/or the platform type in titles, keywords, keywords plus, and abstracts from a total of 2193 studies published between 1990 and 2022. Note that 597 publications within the dataset were not classified into specific sensor categories. These works did not specify a sensor or platform and often employed terms like multispectral or hyperspectral imagery, satellite imagery, satellite, or remote sensing.

RS technologies provide cost‐effective and scalable tools to accurately map and monitor spatial and temporal changes in BCEs over time (Pham et al., 2019), and identify multiple drivers of change (Petus et al., 2014; Richards & Friess, 2016; Thomas et al., 2017). This information can help prioritized and track management actions (Lanceman et al., 2022), as well as to improve the accuracy of estimates of carbon stocks and emissions from BCEs, which is required to achieve climate change goals (Malerba et al., 2023). Historically, aerial photography (*n* = 259 studies) was the first RS tool for mapping BCEs by photointerpretation of RGB tiles, but the development of satellite sensors with lower acquisition costs limited its use over time. Moderate to medium spatial resolution multispectral sensors, such as those onboard Landsat and Sentinel‐2 satellites, can be used to derive regional to global land cover maps and to monitor land cover changes, and multi‐year and seasonal data are freely available (Turner et al., 2015; Willis, 2015). We found that Landsat (30 m resolution; *n* = 587 studies) and Sentinel‐2 (10, 20, and 60 m resolution; *n* = 147 studies), accounting for 34.3% of total studies, were the most common imagery used for the RS of BCEs (Figure 5). However, these optical sensors are unable to capture vegetation structure and bathymetric information; therefore, active sensors like aerial laser scanners (LiDAR) (Maurya et al., 2021; Wang et al., 2019) and remotely operated and autonomous underwater vehicles (Gumusay et al., 2019) have been increasingly used in such studies.

Active sensors (e.g., LiDAR, *n* = 290 studies) have been widely used in BCE studies, mainly to derive structure‐based biophysical parameters such as biomass, canopy height, and vegetation structure, as well as to estimate ground elevation and bathymetric information. The combination of optical imagery and active sensors (i.e., data fusion) has provided the opportunity to improve biomass estimates and vegetation mapping (Salum et al., 2020). The success of active‐passive data fusion relies on the ideal combination of structural‐based (backscatter or height) and chemical‐based (reflectance) information. However, depicting high‐resolution spectral data from satellites and structure information from LiDAR are often expensive, hampering their ample use for experimentation and monitoring at local scales (Farzanmanesh et al., 2021). Therefore, photogrammetric products derived from unmanned aerial vehicles (UAVs) (*n* = 143 studies) and airborne vehicles (*n* = 141 studies) have been used for obtaining optical (i.e., orthomosaics) and structure data (e.g., point‐cloud and digital elevation models) using structure‐from‐motion (SfM) pipelines (e.g., Doughty & Cavanaugh, 2019). The high resolution of UAVs‐ and airborne‐based data products has been used in forest and bog ecosystems to derive ground truth data and upscale vegetation attributes and processes to the landscape level using satellite data (e.g., Bhatnagar et al., 2021; Kattenborn et al., 2019). However, this approach has been used by only a limited number of studies within BCEs (Lanceman et al., 2022).

In more recent years, the use of remotely operated and autonomous underwater vehicles (ROV/AUV; *n* = 143 studies) has also increased. Sensors mounted on remotely operated vehicles have become widespread to derive seafloor data that cannot be retrieved from optical sensors. These active sensors (e.g., side‐scan sonar, multibeam echosounder, and multispectral multibeam echosounder) have opened new avenues for mapping seagrass habitats. However, current data collection, processing, classifications, and validation methodologies are limited to specific species and study areas (Gumusay et al., 2019). BCE mapping and monitoring have also been supported by commercial optical sensors with very high spatial resolution such as IKONOS (<5 m resolution; *n* = 107 studies), Worldview series (1.24–2 m resolution; *n* = 86 studies), and the SPOT series (1.5–20 m resolution; *n* = 66 studies), Although commercial imagery with higher spatial resolution can improve mapping accuracy for BCEs (Belluco et al., 2006; Davranche et al., 2010; Mumby & Edwards, 2002; Thakur et al., 2020), the higher acquisition costs might limit their use in larger study areas or projects with limited funds (Malerba et al., 2023). In contrast, commercial sensors including RapidEye and Hyperion have become less used over time, as they are no longer available.

### 3.5 Keyword network and citation burst

**FIGURE 6:**
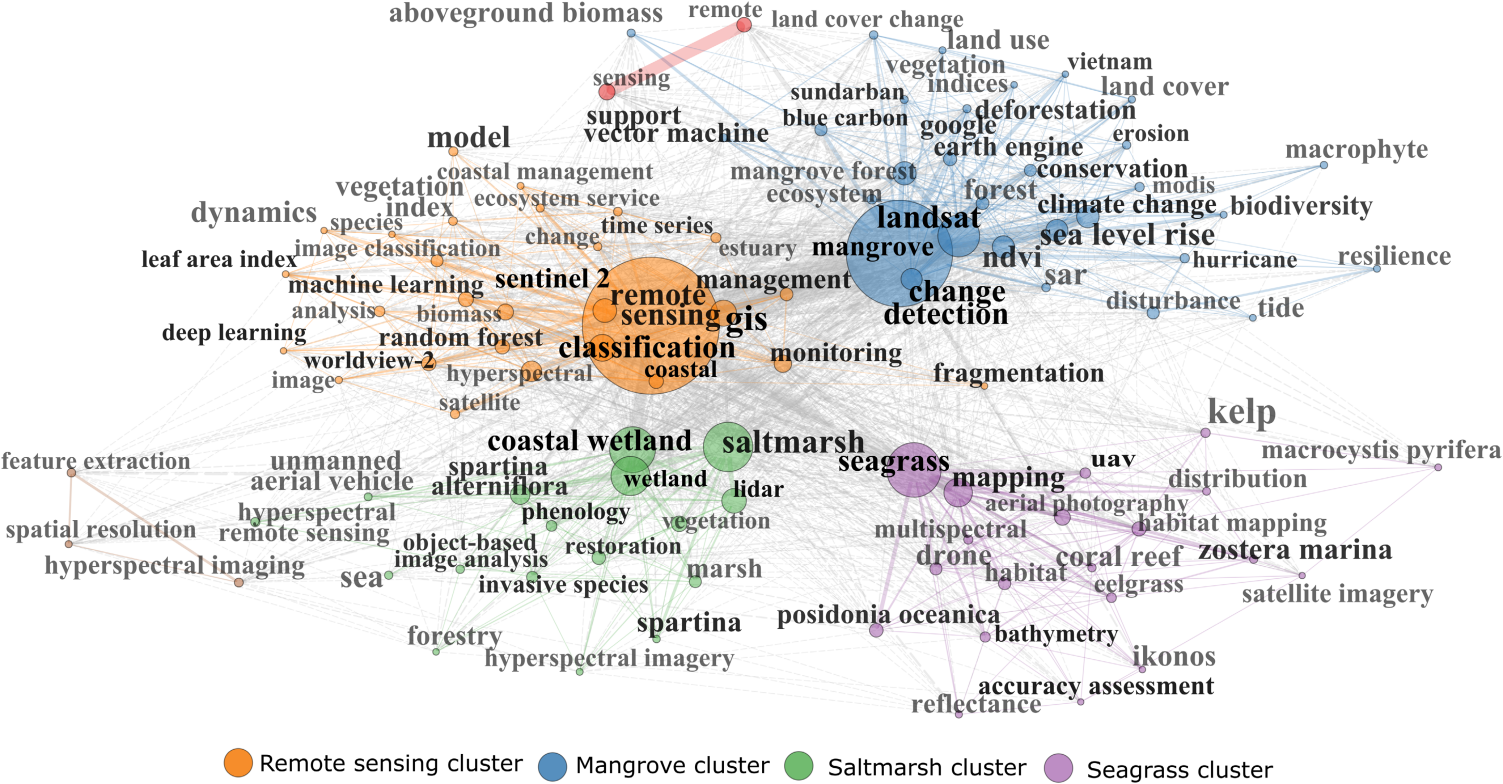
Network map of the co‐occurrence of the top 100 of 4983 keywords in the database. The size of the nodes represents the frequency of the keyword in the network. Larger nodes indicate that these keywords frequently occur in the literature regarding remote sensing and blue carbon ecosystems. The proximity of the nodes indicates how the keywords are related to each other. Lines are connecting nodes with width based on link strength (i.e., the magnitude of their co‐relation). The total strength of the link between a pair of nodes is based on the number of co‐occurrences between keywords represented by those nodes. Nodes highly connected to many other nodes have a higher total strength of links, indicating that these keywords occur frequently in the literature regarding remote sensing and blue carbon ecosystems.

To identify contextual links among publications, we analyzed keyword co‐occurrence (*n* = 100 keywords) among publications. From a total of 4983 keywords, the 10 most used keywords were “remote sensing”, “mangrove”, “seagrass”, “saltmarsh”, “Landsat”, “coastal wetland”, “wetland”, “mapping”, “classification”, and “GIS” (Table S8). Our co‐occurrence network results showed that specific keywords were closely related, indicating that they were often used together in the context of RS applications related to the BCEs field (Figure 6). The terms “remote sensing”, “classification”, “GIS”, “coastal”, “and Sentinel‐2” were strongly related. Additionally, “seagrass” was closely linked to “mapping,” while “mangrove” was closely related to “change detection,” “Landsat,” and “ecosystem.” Finally, “saltmarsh” was closely associated with “coastal wetland,” “wetland,” and “LiDAR.” In our network map, we identified four major clusters of keywords, with remote sensing, mangrove, seagrass, saltmarsh being the center of each cluster and having the highest link strength (Figure 6). The RS cluster (cluster 1, Figure 6) represents publications using multispectral satellite (e.g., Sentinel‐2, Worldview‐2), and hyperspectral imagery for mapping, monitoring, and modeling satellite‐derived vegetation indices (e.g., NDVI) and biophysical parameters (e.g., Leaf Area Index, biomass) by using machine (e.g., random forest, support vector machine) and deep learning algorithms. The mangrove cluster (cluster 2, Figure 6) represents studies using optical satellite imagery (e.g., Landsat, MODIS) and active sensors (e.g., SAR) for land use and land cover change and change detection applications. The saltmarsh cluster (cluster 3, Figure 6) represents studies relying on high‐resolution data (e.g., hyperspectral imagery, LiDAR sensors, UAVs) and advanced image classification techniques, such as object‐based image analysis to map halophyte grass species of the genus *Spartina* (Correll et al., 2019; Gu et al., 2021; Sun et al., 2020; Tian et al., 2020). Finally, the seagrass cluster (cluster 4, Figure 6) represents studies using high‐resolution data (e.g., IKONOS, UAV, bathymetry) to improve mapping accuracies of relevant species of the genus Zostera (e.g., *Zostera marina*) and *Posidonia*.

The cluster analysis highlights the role of RS technologies in assessing the impact of human (e.g., deforestation, species invasion) and natural pressures (e.g., erosion, hurricanes) to support management and conservation efforts in BCEs (e.g., coastal management, conservation, restoration). In addition, we show how the development of new RS technologies (such as LiDAR and UAVs) has been associated with improving ecosystem mapping over time and space (Figure 6). For example, high spatial resolution image data can enhance the study of phenological changes in vegetation at local scales (e.g., canopy height, fractional vegetation cover, aboveground biomass) at lower costs (Grayson et al., 2022) or better deal with high degree of spatial heterogeneity of habitats (e.g., patchiness, fragmentation). With the increasing interest in developing blue carbon restoration projects, improving mapping accuracy at site‐level assessments is key to achieve the precision needed to generate certified carbon credits (e.g., Verra, Australian Emissions Reduction Fund). Moreover, climate change and blue carbon were important research topics in the analysis, which is aligned with current national and international targets (e.g., The UNFCCC's Paris Agreement). However, with the growing development of high‐resolution imagery, advanced techniques (e.g., image fusion, modern artificial intelligence algorithms), and cloud‐based computer systems (such as Google Earth Engine), we expect that RS applications to support climate change and blue carbon‐oriented research will further increase (Malerba et al., 2023). While the main focus has been mangroves, the development and application of such RS tools is relevant to most ecosystem types. This will be essential to effectively monitor and develop up‐to‐date and accurate information in the distribution and conditions of these ecosystems.

**FIGURE 7:**
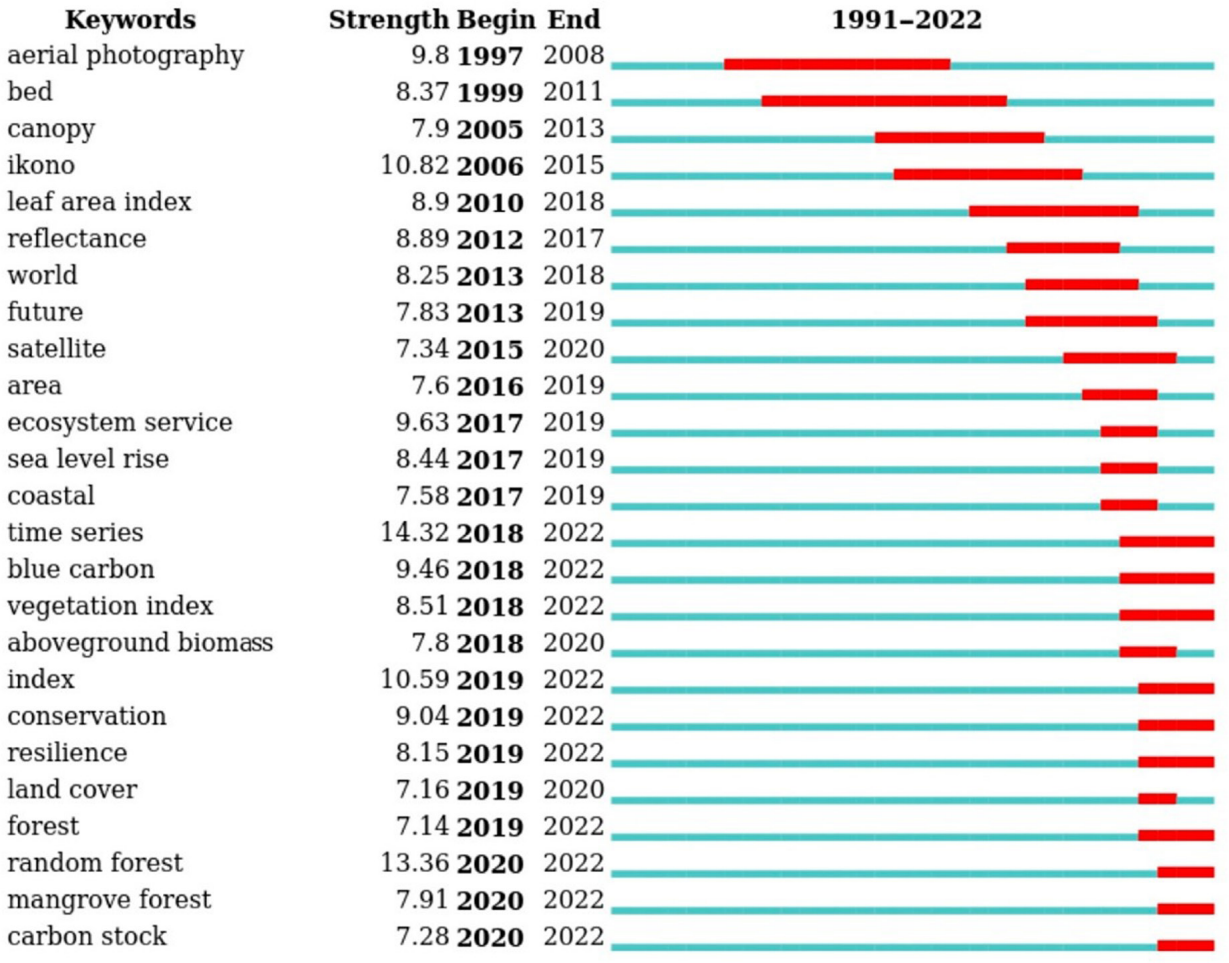
Citation burst analysis of keywords based on the literature review on remote sensing applied to blue carbon science (1990–2022). The strength of the burst represents the impact of the keyword, and the beginning and end years indicate the time range of its influence.

Our citation burst analysis identified emergent topics related to the use of remote sensing for blue carbon research, including estimating aboveground biomass and carbon stocks, and demonstrated that popular topics in the RS field changed over time (Figure 7). In the 1990s, research on aerial photography was popular followed by a transition in the early 2000s toward a focus on biophysical properties (e.g., leaf area index, which is retrieved from satellite‐based vegetation indices) and high spatial resolution sensors such as IKONOS which allowed for higher accuracies for classifying mangrove canopy cover (Wang et al., 2004). In the mid‐2000s, research transitioned toward time series analysis of satellite imagery from satellite missions such as the Landsat series to detect natural and anthropogenic impacts on the extent of BCEs over large geographical areas (Pham et al., 2019; Veettil et al., 2020). Since 2018, popular topics have included time series analysis, land cover mapping, machine learning algorithms, conservation, blue carbon, and carbon stocks. The citation burst analysis suggests a growing interest in understanding the dynamics of coastal wetland vegetation and their responses to environmental and human disturbances. Through this analysis, we were able to identify future research needs including a greater focus on the ecological functions and ecosystem services provided by BCEs, particularly seagrass, saltmarsh, and kelp ecosystems, and their response to human activities and climate change. Additionally, there is a need to develop more advanced models and algorithms, and the integrations of remote sensing with other techniques to address the dynamics and complexity of intertidal environments.

## 4 CONCLUSION

As BCEs are increasingly being recognized as natural climate solutions, countries are increasingly supporting the protection and restoration of these ecosystems to achieve their NDCs under the Paris Agreement. Our findings highlight the various applications of RS technologies to further our understanding of BCEs and their carbon stocks, greenhouse gas fluxes, changes in extent through time, and ecosystem management, including restoration and conservation. Due to their cost‐effectiveness and wide spatial and temporal resolution, RS technologies can play an important role in understanding the factors driving BCEs dynamics and their impacts, supporting countries in achieving their NDCs targets. The synoptic and periodic nature of spaceborne sensors has provided unique opportunities for large‐scale monitoring to detect the change in the extent and condition of BCEs over time and the drivers of these changes. Through this bibliometric analysis, we found that long‐term monitoring of BCE changes, both natural and human‐induced, remains limited to Landsat archives, given its long‐term, open‐access archives. In more recent years, we observed an increased use of object‐based classification techniques and emergent technologies such as UAVs (Cao et al., 2018; Duffy et al., 2018; Thomsen et al., 2019; Ventura et al., 2018). Our results revealed that mangroves received greatest RS attention, and there is a need to increase research efforts toward seagrasses, saltmarshes, and emergent BCEs such as macroalgae (Ortega et al., 2019). Rapid advances in RS technology and the decreasing cost of image acquisition and processing will likely enhance research, management, and restoration of BCEs.

## AUTHOR CONTRIBUTIONS

**Rocio Araya‐Lopez:** Conceptualization (equal); data curation (lead); formal analysis (lead); methodology (lead); visualization (lead); writing – original draft (lead). **Micheli Duarte de Paula Costa:** Conceptualization (equal); supervision (lead); writing – review and editing (lead). **Melissa Wartman:** Conceptualization (equal); supervision (lead); writing – review and editing (lead). **Peter I. Macreadie:** Conceptualization (equal); supervision (lead); writing – review and editing (lead).

## CONFLICT OF INTEREST STATEMENT

The authors declare no competing interests.

## Supporting information


Data S1.

## Data Availability

The data that supported the findings of this study are publicly available at https://doi.org/10.5061/dryad.m0cfxpp8g.
