# Supplementary material for: Trends in the application of remote sensing in blue carbon science
Source: Ecol Evol. 2023 Sep 23;13(9):e10559. doi: 10.1002/ece3.10559 (PMC10517596; doi:10.1002/ece3.10559)
Supplement: Supplementary file 1 — Data S1. [file ECE3-13-e10559-s001.docx]

**Supplementary information**

**Trends in the application of remote sensing in blue carbon science**

Rocio Araya-Lopez^1,*^, Micheli Duarte de Paula Costa^1^, Melissa Wartman^1^, Peter I. Macreadie^1^

^1^ Centre for Integrative Ecology, School of Life and Environmental Sciences, Deakin University, Burwood Campus, Burwood, VIC 3125, Australia

*Corresponding author. *E-mail address*: rarayalopez@deakin.edu.au

**Table S1.** Search string used to find remote sensing and blue carbon related literature in the ISI Web of Science for the time frame between 1900 to 2022.

| **Term** | **Category** | **Search** |
| --- | --- | --- |
| #1 | **Blue carbon ecosystems** | TI= **(**“seagrass*” OR "salt marsh*" OR “saltmarsh*” OR “mangrove*” OR “tidal marsh*" OR “coastal wetland*” or “blue carbon ecosystem*” OR "blue carbon" OR “eelgrass” OR “seaweed” OR “kelp” OR “Asparagopsis” OR “Posidonia” OR “macroalgae” OR “macrophyte*” OR "submerged aquatic vegetation") OR AB= (“seagrass*” OR "salt marsh*" OR “saltmarsh*” OR “mangrove*” OR “tidal marsh*" OR “coastal wetland*” or “blue carbon ecosystem*” OR "blue carbon" OR “eelgrass” OR “seaweed” OR “kelp” OR “Asparagopsis” OR “Posidonia” OR “macroalgae” OR “macrophyte*” OR "submerged aquatic vegetation") OR AK= (“seagrass*” OR "salt marsh*" OR “saltmarsh*” OR “mangrove*” OR “tidal marsh*" OR “coastal wetland*” or “blue carbon ecosystem*” OR "blue carbon" OR “eelgrass” OR “seaweed” OR “kelp” OR “Asparagopsis” OR “Posidonia” OR “macroalgae” OR “macrophyte*” OR "submerged aquatic vegetation") OR KP= (“seagrass*” OR "salt marsh*" OR “saltmarsh*” OR “mangrove*” OR “tidal marsh*" OR “coastal wetland*” or “blue carbon ecosystem*” OR "blue carbon" OR “eelgrass” OR “seaweed” OR “kelp” OR “Asparagopsis” OR “Posidonia” OR “macroalgae” OR “macrophyte*” OR "submerged aquatic vegetation"). |
| #2 | **Remote sensing** | TI= (“remote sensing” OR “Imagery” OR “multispectral” OR "hyperspectral" OR "radar" OR "LIDAR” OR "UAV*” OR “unmanned aerial vehicle" OR "airborne" OR "satellite image*" OR "satellite"OR "Landsat*" OR "Sentinel*" OR "worldview*" OR "SPOT" OR "IKONOS" OR "Quickbird" OR "Geo-Eye*" OR "aerial photograph*" OR " digital image*" OR "Remotely Operated Vehicle*" OR " Autonomous Underwater Vehicle*" OR "sensor" "Vessel" OR " Acoustic") OR AB= (“remote sensing” OR “Imagery” OR “multispectral” OR "hyperspectral" OR "radar" OR "LIDAR” OR "UAV*” OR “unmanned aerial vehicle" OR "airborne" OR "satellite image*" OR "satellite"OR "Landsat*" OR "Sentinel*" OR "worldview*" OR "SPOT" OR "IKONOS" OR "Quickbird" OR "Geo-Eye*" OR "aerial photograph*" OR " digital image*" OR "Remotely Operated Vehicle*" OR " Autonomous Underwater Vehicle*" OR "sensor" "Vessel" OR " Acoustic") OR AK= (“remote sensing” OR “Imagery” OR “multispectral” OR "hyperspectral" OR "radar" OR "LIDAR” OR "UAV*” OR “unmanned aerial vehicle" OR "airborne" OR "satellite image*" OR "satellite"OR "Landsat*" OR "Sentinel*" OR "worldview*" OR "SPOT" OR "IKONOS" OR "Quickbird" OR "Geo-Eye*" OR "aerial photograph*" OR " digital image*" OR "Remotely Operated Vehicle*" OR " Autonomous Underwater Vehicle*" OR "sensor" "Vessel" OR " Acoustic") OR KP= (“remote sensing” OR “Imagery” OR “multispectral” OR "hyperspectral" OR "radar" OR "LIDAR” OR "UAV*” OR “unmanned aerial vehicle" OR "airborne" OR "satellite image*" OR "satellite"OR "Landsat*" OR "Sentinel*" OR "worldview*" OR "SPOT" OR "IKONOS" OR "Quickbird" OR "Geo-Eye*" OR "aerial photograph*" OR " digital image*" OR "Remotely Operated Vehicle*" OR " Autonomous Underwater Vehicle*" OR "sensor" "Vessel" OR " Acoustic"). |

**Table S2.** Inclusion criteria used for title and abstract screening.

| **Criteria** | **References** |
| --- | --- |
| Mapping, monitoring, and measuring biophysical parameters of coastal wetlands or at least partially on one or several BCEs | Mondal, I., Thakur, S., Juliev, M., & Kumar De, T. (2021). Comparative analysis of forest canopy mapping methods for the Sundarban biosphere reserve, West Bengal, India. *Environment, Development and Sustainability*, *23*(10), 15157–15182.  <https://doi.org/10.1007/s10668-021-01291-6>  Durgan, S. D., Zhang, C., Duecaster, A., Fourney, F., & Su, H. (n.d.). *Unmanned Aircraft System Photogrammetry for Mapping Diverse Vegetation Species in a Heterogeneous Coastal Wetland*. <https://doi.org/10.1007/s13157-020-01373-7/Published>  Mumby, P. J., & Edwards, A. J. (n.d.). *Mapping marine environments with IKONOS imagery: enhanced spatial resolution can deliver greater thematic accuracy*. www.elsevier.com/locate/rse |
| Assessing land use (e.g., deforestation, reclamation for agriculture and aquaculture, non-native species) impacts on coastal wetland or BCEs | Aslan, A., Rahman, A. F., Robeson, S. M., & Ilman, M. (2021). Land-use dynamics associated with mangrove deforestation for aquaculture and the subsequent abandonment of ponds. *Science of the Total Environment*, *791*. <https://doi.org/10.1016/j.scitotenv.2021.148320>  Mansaray, L. R., Huang, J., & Kamara, A. A. (2016). Mapping deforestation and urban expansion in Freetown, Sierra Leone, from pre- to post-war economic recovery. *Environmental Monitoring and Assessment*, *188*(8). <https://doi.org/10.1007/s10661-016-5469-y>  Disperati, L., & Virdis, S. G. P. (2015). Assessment of land-use and land-cover changes from 1965 to 2014 in Tam Giang-Cau Hai Lagoon, central Vietnam. *Applied Geography*, *58*, 48–64. <https://doi.org/10.1016/j.apgeog.2014.12.012> |
| Assessing coastal hazard (e.g., oil spill, storm surge, erosion, flooding, sea level rise) and invasive species on coastal wetland or BCEs | Fu, S., Zheng, S., Gao, W., Wang, A., Ma, X., Sun, L., Sun, T., & Shao, D. (2021). Effects of the Water-Sediment Regulation Scheme on the Expansion of Spartina alterniflora at the Yellow River Estuary, China. *Frontiers in Environmental Science*, *9*. <https://doi.org/10.3389/fenvs.2021.642442>  Turner, R. E., McClenachan, G., & Tweel, A. W. (2016). Islands in the oil: Quantifying salt marsh shoreline erosion after the Deepwater Horizon oiling. *Marine Pollution Bulletin*, *110*(1), 316–323. h<ttps://doi.org/10.1016/j.marpolbul.2016.06.046>  Doyle, T. W., Krauss, K. W., & Wells, C. J. (2009). Landscape analysis and pattern of hurricane impact and circulation on mangrove forests of the Everglades. *Wetlands*, *29*, 44–53. |
| Hydro-geomorphological processes link to vegetated coastal habitats | Author, B., Souza-Filho, P., Gonçalves, F., Rodrigues, S., Costa, F., & Miranda, F. (2009). Multi-Sensor Data Fusion for Geomorphological and Environmental Sensitivity Index Mapping in the Amazonian Mangrove Coast, Brazil. In *Source: Journal of Coastal Research: Vol. II* (Issue 56).  Chen, C., Zhang, C., Schwarz, C., Tian, B., Jiang, W., Wu, W., Garg, R., Garg, P., Aleksandr, C., Mikhail, S., & Zhou, Y. (2022). Mapping three-dimensional morphological characteristics of tidal salt-marsh channels using UAV structure-from-motion photogrammetry. *Geomorphology*, *407*. <https://doi.org/10.1016/j.geomorph.2022.108235>  Hou, W., Zhang, R., Xi, Y., Liang, S., & Sun, Z. (2020). The role of waterlogging stress on the distribution of salt marsh plants in the Liao River estuary wetland. *Global Ecology and Conservation*, *23*. <https://doi.org/10.1016/j.gecco.2020.e01100>  Ashall, L. M., Mulligan, R. P., van Proosdij, D., & Poirier, E. (2016). Application and validation of a three-dimensional hydrodynamic model of a macrotidal salt marsh. *Coastal Engineering*, *114*, 35–46. <https://doi.org/10.1016/j.coastaleng.2016.04.005> |
| Soil properties (e.g., salinity, bulk density) of BCEs | Anne, N. J. P., Abd-Elrahman, A. H., Lewis, D. B., & Hewitt, N. A. (2014). Modeling soil parameters using hyperspectral image reflectance insubtropical coastal wetlands. *International Journal of Applied Earth Observation and Geoinformation*, *33*(1), 47–56. https://doi.org/10.1016/j.jag.2014.04.007  Chi, Y., Sun, J., Liu, W., Wang, J., & Zhao, M. (2019). Mapping coastal wetland soil salinity in different seasons using an improved comprehensive land surface factor system. *Ecological Indicators*, *107*. <https://doi.org/10.1016/j.ecolind.2019.105517> |
| Testing or improving RS approaches in BCEs | Younes, N., Northfield, T. D., Joyce, K. E., Maier, S. W., Duke, N. C., & Lymburner, L. (2020). A novel approach to modelling mangrove phenology from satellite images: A case study from Northern Australia. *Remote Sensing*, *12*(24), 1–24.  <https://doi.org/10.3390/rs12244008>  Cho, H. J., Piñeyro, B., & Gasdia, F. W. (2016). Water correction for improved benthic vegetation signal using satellite-borne hyperspectral data. *International Journal of Remote Sensing*, *37*(17), 4084–4100.  <https://doi.org/10.1080/01431161.2016.1207262>  Zanella, R., Boccacci, P., Zanni, L., & Bertero, M. (2009). Efficient gradient projection methods for edge-preserving removal of Poisson noise. *Inverse Problems*, *25*(4). <https://doi.org/10.1088/0266-5611/25/4/045010> |
| Reviewing RS applications on coastal wetlands or BCEs | Gumusay, M. U., Bakirman, T., Kizilkaya, I. T., & Onur, N. (2019). A review of seagrass detection , mapping and monitoring applications using acoustic systems. *European Journal of Remote Sensing*, *52*(1), 1–29. <https://doi.org/10.1080/22797254.2018.1544838>  Pham, T., Yokoya, N., Bui, D. T., Yoshino, K., & Friess, D. A. (2019). Remote Sensing Approaches for Monitoring Mangrove Species, Structure, and Biomass: Opportunities and Challenges. *REMOTE SENSING*, *11*(3). <https://doi.org/10.3390/rs11030230> |

**Table S3.** Classification of journals by the number of articles referencing remote sensing applied to blue carbon science for the period 1990-2022.

| **Number of articles per journal** | **Number of journals** | **Percentage of the number of journals (%)** | **Number of articles** | **Percentage of the number of articles (%)** |
| --- | --- | --- | --- | --- |
| 1 | 173 | 48.05 | 173 | 7.8 |
| 2-9 | 143 | 39.72 | 564 | 25.7 |
| 10-30 | 34 | 9.4 | 571 | 26.03 |
| 31-90 | 5 | 1.38 | 199 | 9.03 |
| 91-262 | 5 | 1.38 | 686 | 31.2 |

**Table S4.** Top journals with the highest impact factor publishing studies referencing remote sensing applied to blue carbon science for the period 1990-2022.

| **Journal** | **Impact factor** | **Reference** |
| --- | --- | --- |
| Science | 63.832 | Halpern, B. S., K. Cottenie, and B. R. Broitman. 2006. ‘Strong Top-down Control in Southern California Kelp Forest Ecosystems’. SCIENCE 312(5777):1230–32. doi: 10.1126/science.1128613. |
| Nature Climate Change | 28.862 | Arias-Ortiz, Ariane, Oscar Serrano, Pere Masqué, Paul S. Lavery, Ute Mueller, Gary A. Kendrick, Mohammad Rozaimi, Alba Esteban, James W. Fourqurean, Núria Marbà, Miquel-Angel Mateo, Kathy Murray, Michael J. Rule, and Carlos M. Duarte. 2018. ‘A Marine Heatwave Drives Massive Losses from the World’s Largest Seagrass Carbon Stocks’. Nature Climate Change. |
| Nature Sustainability | 27.157 | Wang, Xinxin, Xiangming Xiao, Xiao Xu, Zhenhua Zou, Bangqian Chen, Yuanwei Qin, Xi Zhang, Jinwei Dong, Diyou Liu, Lianghao Pan, and Bo Li. 2021. ‘Rebound in China’s Coastal Wetlands Following Conservation and Restoration’. NATURE SUSTAINABILITY 4(12):1076+. doi: 10.1038/s41893-021-00793-5. |
| Nature Communications | 17.694 | Lagomasino, David, Temilola Fatoyinbo, Edward Castaneda-Moya, Bruce D. Cook, Paul M. Montesano, Christopher S. R. Neigh, Lawrence A. Corp, Lesley E. Ott, Selena Chavez, and Douglas C. Morton. 2021. ‘Storm Surge and Ponding Explain Mangrove Dieback in Southwest Florida Following Hurricane Irma’. NATURE COMMUNICATIONS 12(1). doi: 10.1038/s41467-021-24253-y. |

**Table S5.** The top 10 most cited articles referencing remote sensing applied to blue carbon science found in the ISI Web of Science for the period 1990-2022. Here, we have also included the number of citations for each paper found in Google Scholar and Wos by April 2023.

| **Ranking** | **WoS** | **Google Scholar** | **Reference** |
| --- | --- | --- | --- |
| 1 | 1503 | 3074 | Giri, C., Ochieng, E., Tieszen, L. L., Zhu, Z., Singh, A., Loveland, T., Masek, J., & Duke, N. (2011). Status and distribution of mangrove forests of the world using earth observation satellite data. *Global Ecology and Biogeography*, *20*(1), 154–159.  <https://doi.org/10.1111/j.1466-8238.2010.00584.x> |
| 2 | 522 | 795 | Tyberghein, L., Verbruggen, H., Pauly, K., Troupin, C., Mineur, F., & de Clerck, O. (2012). Bio-ORACLE: A global environmental dataset for marine species distribution modelling. *Global Ecology and Biogeography*, *21*(2), 272–281.  <https://doi.org/10.1111/j.1466-8238.2011.00656.x> |
| 3 | 502 | 842 | Richards, D. R., & Friess, D. A. (2016). Rates and drivers of mangrove deforestation in Southeast Asia, 2000-2012. *Proceedings of the National Academy of Sciences of the United States of America*, *113*(2), 344–349. <https://doi.org/10.1073/pnas.1510272113> |
| 4 | 475 | 809 | Hamilton, S. E., & Casey, D. (2016). Creation of a high spatio-temporal resolution global database of continuous mangrove forest cover for the 21st century (CGMFC-21). *Global Ecology and Biogeography*, *25*(6), 729–738. <https://doi.org/10.1111/geb.12449> |
| 5 | 413 | 783 | Schmidt, K. S., & Skidmore, a. K. (2003). Spectral discrimination of vegetation types in a coastal wetland. *Remote Sensing of Environment*, *85*(1), 92–108. <https://doi.org/10.1016/S0034-4257(02)00196-7> |
| 6 | 379 | 708 | Kuenzer, C., Bluemel, A., Gebhardt, S., Quoc, T. V., & Dech, S. (2011). Remote Sensing of Mangrove Ecosystems: A Review. *REMOTE SENSING*, *3*(5), 878–928. <https://doi.org/10.3390/rs3050878> |
| 7 | 347 | 519 | Cavanaugh, K. C., Kellner, J. R., Forde, A. J., Gruner, D. S., Parker, J. D., Rodriguez, W., & Feller, I. C. (2014). Poleward expansion of mangroves is a threshold response to decreased frequency of extreme cold events. *Proceedings of the National Academy of Sciences of the United States of America*, *111*(2), 723–727.  <https://doi.org/10.1073/pnas.1315800111> |
| 8 | 295 | 563 | Wang, L., Sousa, W. P., Gong, P., & Biging, G. S. (2004). Comparison of IKONOS and QuickBird images for mapping mangrove species on the Caribbean coast of Panama. *REMOTE SENSING OF ENVIRONMENT*, *91*(3–4), 432–440. <https://doi.org/10.1016/j.rse.2004.04.005> |
| 9 | 252 | 694 | Macleod, R. D., & Congalton, R. G. (1998). A Quantitative Comparison of Change-Detection Algorithms for Monitoring Eelgrass from Remotely Sensed Data. *Photogrammetric Engineering and Remote Sensing*, *64*, 207–216. |
| 10 | 266 | 478 | Zhao, B., Kreuter, U., Li, B., Ma, Z., Chen, J., & Nakagoshi, N. (2004). An ecosystem service value assessment of land-use change on Chongming Island, China. *Land Use Policy*, *21*(2), 139–148.  <https://doi.org/10.1016/j.landusepol.2003.10.003> |

**Table S6.** The top 10 most cited articles in the last 5 years referencing remote sensing applied to blue carbon science found in the ISI Web of Science for the period 1990-2022. Here, we have also included the number of citations for each paper found in Google Scholar and WoS by April 2023.

| **Ranking** | **WoS** | **Google Scholar** | **Reference** |
| --- | --- | --- | --- |
| 1 | 280 | 421 | Bunting, P., Rosenqvist, A., Lucas, R. M., Rebelo, L. M., Hilarides, L., Thomas, N., Hardy, A., Itoh, T., Shimada, M., & Finlayson, C. M. (2018). The global mangrove watch - A new 2010 global baseline of mangrove extent. *Remote Sensing*, *10*(10). <https://doi.org/10.3390/rs10101669> |
| 2 | 208 | 307 | Arias-Ortiz, A., Serrano, O., Masqué, P., Lavery, P. S., Mueller, U., Kendrick, G. A., Rozaimi, M., Esteban, A., Fourqurean, J. W., Marbà, N., Mateo, M. A., Murray, K., Rule, M. J., & Duarte, C. M. (2018). A marine heatwave drives massive losses from the world’s largest seagrass carbon stocks. *Nature Climate Change*, *8*(4), 338–344. <https://doi.org/10.1038/s41558-018-0096-y> |
| 3 | 153 | 226 | Sanderman, J., Hengl, T., Fiske, G., Solvik, K., Adame, M. F., Benson, L., Bukoski, J. J., Carnell, P., Cifuentes-Jara, M., Donato, D., Duncan, C., Eid, E. M., Ermgassen, P. Z., Lewis, C. J. E., Macreadie, P. I., Glass, L., Gress, S., Jardine, S. L., Jones, T. G., … Landis, E. (2018). A global map of mangrove forest soil carbon at 30 m spatial resolution. *Environmental Research Letters*, *13*(5). <https://doi.org/10.1088/1748-9326/aabe1c> |
| 4 | 210 | 362 | Goldberg, L., Lagomasino, D., Thomas, N., & Fatoyinbo, T. (2020). Global declines in human‐driven mangrove loss - Goldberg - 2020 - Global Change Biology - Wiley Onli. *Global Change Biology* , *26*, 5844–5855. <https://doi.org/10.1111/gcb.15275> |
| 5 | 126 | 203 | Cao, J., Leng, W., Liu, K., Liu, L., He, Z., & Zhu, Y. (2018). Object-Based mangrove species classification using unmanned aerial vehicle hyperspectral images and digital surface models. *Remote Sensing*, *10*(1). <https://doi.org/10.3390/rs10010089> |
| 6 | 134 | 204 | Wang, L., Jia, M., Yin, D., & Tian, J. (2019). A review of remote sensing for mangrove forests: 1956-2018. *REMOTE SENSING OF ENVIRONMENT*, *231*. <https://doi.org/10.1016/j.rse.2019.111223> |
| 7 | 113 | 170 | Thomsen, M. S., Mondardini, L., Alestra, T., Gerrity, S., Tait, L., South, P. M., Lilley, S. A., & Schiel, D. R. (2019). Local Extinction of bull kelp (Durvillaea spp.) due to a marine heatwave. *Frontiers in Marine Science*, *6*(MAR). <https://doi.org/10.3389/fmars.2019.00084> |
| 8 | 110 | 147 | Pastor-Guzman, J., Dash, J., & Atkinson, P. M. (2018). Remote sensing of mangrove forest phenology and its environmental drivers. *Remote Sensing of Environment*, *205*, 71–84. <https://doi.org/10.1016/j.rse.2017.11.009> |
| 9 | 110 | 161 | Ventura, D., Bonifazi, A., Gravina, M. F., Belluscio, A., & Ardizzone, G. (2018). Mapping and classification of ecologically sensitive marine habitats using unmanned aerial vehicle (UAV) imagery and Object-Based Image Analysis (OBIA). *Remote Sensing*, *10*(9). <https://doi.org/10.3390/rs10091331> |
| 10 | 94 | 146 | Duffy, J. P., Pratt, L., Anderson, K., Land, P. E., & Shutler, J. D. (2018). Spatial assessment of intertidal seagrass meadows using optical imaging systems and a lightweight drone. *Estuarine, Coastal and Shelf Science*, *200*, 169–180. <https://doi.org/10.1016/j.ecss.2017.11.001> |

**Table S7.** List of countries within geographic regions publishing remote sensing articles applied to blue carbon ecosystems based on corresponding-author affiliations per habitat type.

| **Geographic region** | **Countries** |
| --- | --- |
| North America | USA, Canada, USA, Mexico, Panama. |
| South America | Brazil, Argentina, Chile, Colombia, Ecuador, Uruguay, Venezuela, French Guiana. |
| Europe | Belgium, Bulgaria, Croatia, Denmark, Estonia, Finland, France, Germany, Greece, Ireland, Italy, Malta, Lithuania, Monaco, Netherlands, Norway, Poland, Portugal, Romania, Russia, Slovenia, Spain, Sweden, Switzerland, UK. |
| Africa | Benin, Botswana, Cameroon, Gambia, Ghana, Kenya, Madagascar, Morocco, Mozambique, Nigeria, South Africa, Egypt. |
| Middle East | Bahrain, Kuwait, Qatar, Saudi Arabia, U Arab Emirates, Iran, Torquay. |
| South Asia | India, Bangladesh, Pakistan, Sri Lanka |
| Southeast Asia | Indonesia, Malaysia, Myanmar, Philippines, Singapore, Thailand, Vietnam. |
| East Asia | China, Japan, South Korea, Taiwan. |
| Oceania | Australia, New Zealand, Papua New Guinea, New Caledonia. |
| Caribbean | Bermuda, Cayman Island, Dominca, Jamaica, Trinidad Tobago |


**Table S8.** Top 10 keywords ranked according to the total link strength.

| **Ranking** | **Keyword** | **Occurrences** | **Total link strength** |
| --- | --- | --- | --- |
| **1** | Remote sensing | 482 | 3066 |
| **2** | Mangrove | 395 | 2443 |
| **3** | Seagrass | 173 | 1096 |
| **4** | Saltmarsh | 164 | 888 |
| **5** | Landsat | 124 | 848 |
| **6** | Coastal wetland | 122 | 792 |
| **7** | Wetland | 101 | 710 |
| **8** | Mapping | 82 | 543 |
| **9** | Classification | 72 | 479 |
| **10** | GIS | 67 | 434 |

**Table S9.** Remote sensing applications, remote sensing type, and variables measured for blue carbon ecosystems.

| **RS system** | | **Passive** | | | | | | | **Active** | | |
| --- | --- | --- | --- | --- | --- | --- | --- | --- | --- | --- | --- |
| **Platform** | | Satellite | | | | Aerial Photography | Airborne | UAV | Satellite | Airborne | Underwater |
| **Sensor type** | | Multi-spectral  (LSR)^1^ | Multi-spectral  (MSR)^2^ | Multi-  spectral  (HSR)^3^ | Hyper-spectral | - | Hyper-spectral | RGB, multispectral and hyperspectral | Synthetic aperture radar | Laser | Acoustic |
| **Example of sensor** | | MODIS | Landsat, Sentinel-2, SPOT | QuickBird, IKONOS, Worldview | Hyperion | - | CASI, HyMap, AISA | Digital camera | RADAR-SAT, Sentinel-1, ALOS- PALSAR | LiDAR | - |
| **Applications of RS in BCEs** | **Variables** |  |  |  |  |  |  |  |  |  |  |
| **Mapping** | **Land use and land cover change** | [9, 8] | [14, 15, 16, 40, 41] | [51, 66, 70] |  | [87, 88, 89, 90, 91] |  |  |  |  | [175, 176] |
|  | **Presence-Absence** |  |  |  |  |  |  |  |  |  | [167] |
|  | **Vegetation cover** | [2] | [18, 19, 21, 45] | [52, 53, 55] | [71] | [83, 84, 85, 86] |  | [104, 105, 113, 115, 117] | [141, 142, 143, 144, 145] | [152, 153] | [168, 169, 178, 179] |
| **Biodiversity** | **Community composition** |  | [17, 20] | [58, 65] | [72, 74] | [92, 95] | [123, 125] | [118] |  |  | [174, 180] |
|  | **Species discrimination** |  | [165] | [54, 59, 64] | [73, 75, 76] | [93] | [124, 128, 129] | [106, 114, 120, 121] |  |  | [177] |
| **Vegetation structure** | **Above-ground biomass** |  | [24, 37, 49] | [56, 62] | [82] |  |  | [107, 110, 111] | [146, 147, 148, 149, 150] | [151] |  |
|  | **Canopy height** |  | [23] |  |  |  |  | [122] | [136, 137, 138, 162] | [155, 159, 163, 164] | [172] |
|  | **Canopy and crown** |  | [25] | [69, 154, 161] |  | [160] |  | [102, 122] |  | [163, 164] |  |
|  | **Tree density** |  | [38] |  |  |  |  |  | [139, 140] |  |  |
|  | **Basal area** |  |  |  |  |  |  |  | [139, 140] |  |  |
| **Biophysical parameter** | **LAI** | [12] | [22] | [61, 63] | [78] |  |  | [108] | [139, 140], 162 |  |  |
|  | **Leaf Chlorophyl, pigments** | [13] | [27] |  | [77] |  | [127] | [103] |  |  |  |
|  | **Dry matter, water content, nutrients** |  | [26] |  | [81] |  |  |  |  |  |  |
| **Vegetation functions** | **Productivity** | [6] | [39] |  |  |  |  |  |  |  |  |
|  | **Oxygen production** |  |  |  |  |  |  |  |  |  | [170, 171, 173] |
|  | **Phenology** | [1, 5, 10] | [35,36] |  |  |  |  |  |  |  |  |
| **Disturbance** | **Pollution (land-based, oil spill)** | [7] | [28, 29] |  |  | [94, 101] | [126, 130] |  |  |  |  |
|  | **Flooding** | [4] | [46,47, 48] |  |  | [96, 97] |  |  |  |  |  |
|  | **Cyclones, typhoons, hurricanes** |  | [33, 34] | [57] |  |  |  | [109, 119] |  |  |  |
|  | **Temperature** |  |  | [60] |  |  |  |  |  |  |  |
|  | **Invasion** |  |  | [83] | [79] | [98] |  | [112] |  |  |  |
|  | **Recovery** | [3] | [30, 31, 32] | [67, 68] |  | [99, 100, 101] | [131, 132] | [116, 119] |  |  |  |
| **Soil** | **Soil properties** |  | [42, 43, 44] |  | [80] |  |  |  | [133, 134, 135] |  |  |
|  | **Topography and bathymetry** |  |  |  |  |  |  |  |  | [156, 157, 158, 166] |  |

^1^LSR: Low spatial resolution

^2^ MSR: Moderate spatial resolution

^3^ HSR: High spatial resolution

**
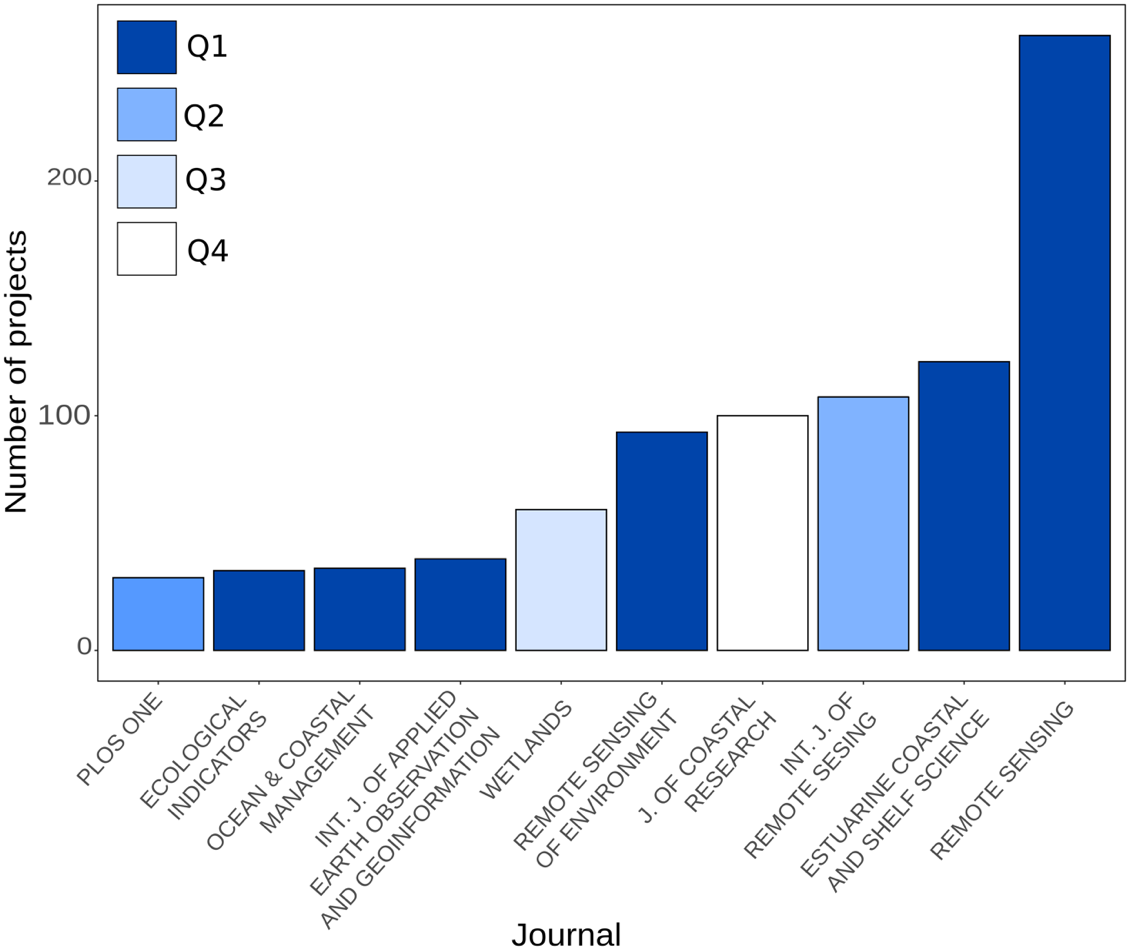
**

**Figure S1.** Top 10 journals publishing studies referencing remote sensing applied to blue carbon science for the period 1990-2022. Journal in ascended order, Remote Sensing, Estuarine Coastal and Shelf Science, International Journal of Remote Sensing, Journal of Coastal Research, Remote Sensing of Environment, Wetlands, International Journal of Applied Earth Observation and Geoinformation, Ocean and Coastal Management, Ecological Indicators, Plos One.

**References**

[1] [A. F. Rahman, D. Dragoni, K. Didan, A. Barreto-Munoz, and J. A. Hutabarat, “Detecting large scale conversion of mangroves to aquaculture with change point and mixed-pixel analyses of high-fidelity MODIS data,” *Remote Sens. Environ.*, vol. 130, pp. 96–107, Mar. 2013, doi:](http://paperpile.com/b/I9CICW/Aj49) [10.1016/j.rse.2012.11.014.](http://dx.doi.org/10.1016/j.rse.2012.11.014.)

[2] [K. Yu and C. Hu, “Long-term vegetation changes in four types of wetland in China and USA between 2000 and 2011: observations from MODIS,” *Int. J. Remote Sens.*, vol. 40, no. 11, pp. 4302–4325, Jun. 2019, doi:](http://paperpile.com/b/I9CICW/g1x1) [10.1080/01431161.2018.1562584.](http://dx.doi.org/10.1080/01431161.2018.1562584.)

[3] [M. J. Faruque *et al.*, “Monitoring of land use and land cover changes by using remote sensing and GIS techniques at human-induced mangrove forests areas in Bangladesh,” *Remote Sensing Applications: Society and Environment*, vol. 25, p. 100699, Jan. 2022, doi:](http://paperpile.com/b/I9CICW/WHgS) [10.1016/j.rsase.2022.100699.](http://dx.doi.org/10.1016/j.rsase.2022.100699.)

[4] [A. Ruiz-Luna and C. A. Berlanga-Robles, “Land use, land cover changes and coastal lagoon surface reduction associated with urban growth in northwest Mexico,” *Landsc. Ecol.*, vol. 18, no. 2, pp. 159–171, Mar. 2003, doi:](http://paperpile.com/b/I9CICW/Mwrb) [10.1023/A:1024461215456.](http://dx.doi.org/10.1023/A:1024461215456.)

[5] [H. T. Le *et al.*, “Characterizing Spatiotemporal Patterns of Mangrove Forests in Can Gio Biosphere Reserve Using Sentinel-2 Imagery,” *NATO Adv. Sci. Inst. Ser. E Appl. Sci.*, vol. 10, no. 12, p. 4058, Jun. 2020, doi:](http://paperpile.com/b/I9CICW/wIoz) [10.3390/app10124058.](http://dx.doi.org/10.3390/app10124058.)

[6] [M. S. Hossain, M. Hashim, J. S. Bujang, M. H. Zakaria, and A. M. Muslim, “Assessment of the impact of coastal reclamation activities on seagrass meadows in Sungai Pulai estuary, Malaysia, using Landsat data (1994–2017),” *Int. J. Remote Sens.*, vol. 40, no. 9, pp. 3571–3605, May 2019, doi:](http://paperpile.com/b/I9CICW/dcXC) [10.1080/01431161.2018.1547931.](http://dx.doi.org/10.1080/01431161.2018.1547931.)

[7] [B. Tian, W. Wu, Z. Yang, and Y. Zhou, “Drivers, trends, and potential impacts of long-term coastal reclamation in China from 1985 to 2010,” *Estuar. Coast. Shelf Sci.*, vol. 170, pp. 83–90, Mar. 2016, doi:](http://paperpile.com/b/I9CICW/RubJ) [10.1016/j.ecss.2016.01.006.](http://dx.doi.org/10.1016/j.ecss.2016.01.006.)

[8] [C. A. Johnston, T. Watson, and P. T. Wolter, “Sixty-three Years of Land Alteration in Erie Township,” *J. Great Lakes Res.*, vol. 33, pp. 253–268, Jan. 2007, doi:](http://paperpile.com/b/I9CICW/DraT) [10.3394/0380-1330(2007)33[253:SYOLAI]2.0.CO;2.](http://dx.doi.org/10.3394/0380-1330(2007)33%5B253:SYOLAI%5D2.0.CO;2.)

[9] [D. Traganos and P. Reinartz, “Interannual Change Detection of Mediterranean Seagrasses Using RapidEye Image Time Series,” *Front. Plant Sci.*, vol. 9, p. 96, Feb. 2018, doi:](http://paperpile.com/b/I9CICW/K8cC) [10.3389/fpls.2018.00096.](http://dx.doi.org/10.3389/fpls.2018.00096.)

[10] [S. N. Hayashi, P. W. M. Souza-Filho, W. R. Nascimento Jr, and M. E. B. Fernandes, “The effect of anthropogenic drivers on spatial patterns of mangrove land use on the Amazon coast,” *PLoS One*, vol. 14, no. 6, p. e0217754, Jun. 2019, doi:](http://paperpile.com/b/I9CICW/OdRx) [10.1371/journal.pone.0217754.](http://dx.doi.org/10.1371/journal.pone.0217754.)

[11] [R. S. Fletcher, W. Pulich, and B. Hardegree, “A Semiautomated Approach for Monitoring Landscape Changes in Texas Seagrass Beds from Aerial Photography,” *J. Coast. Res.*, vol. 25, no. 2 (252), pp. 500–506, Mar. 2009, doi:](http://paperpile.com/b/I9CICW/8tl5) [10.2112/07-0882.1.](http://dx.doi.org/10.2112/07-0882.1.)

[12] [B. Baily and R. Inkpen, “Assessing historical saltmarsh change; an investigation into the reliability of historical saltmarsh mapping using contemporaneous aerial photography and cartographic data,” *J. Coast. Conserv.*, vol. 17, no. 3, pp. 503–514, Sep. 2013, doi:](http://paperpile.com/b/I9CICW/5TFb) [10.1007/s11852-013-0250-7.](http://dx.doi.org/10.1007/s11852-013-0250-7.)

[13] [L. R. Hernández-Cruz, S. J. Purkis, and B. M. Riegl, “Documenting decadal spatial changes in seagrass and Acropora palmata cover by aerial photography analysis in Vieques, Puerto Rico: 1937--2000,” *Bull. Mar. Sci.*, vol. 79, no. 2, pp. 401–414, 2006, [Online]. Available:](http://paperpile.com/b/I9CICW/fYVA) <https://www.ingentaconnect.com/content/umrsmas/bullmar/2006/00000079/00000002/art00012>

[14] [F. Dahdouh-Guebas, A. Verheyden, W. De Genst, S. Hettiarachchi, and N. Koedam, “Four decade vegetation dynamics in Sri Lankan mangroves as detected from sequential aerial photography: A case study in Galle,” *Bull. Mar. Sci.*, vol. 67, no. 2, pp. 741–759, 2000, [Online]. Available:](http://paperpile.com/b/I9CICW/Wtbw) <https://www.ingentaconnect.com/content/umrsmas/bullmar/2000/00000067/00000002/art00005>

[15] [B. D. Robbins, “Quantifying temporal change in seagrass areal coverage: the use of GIS and low resolution aerial photography,” *Aquat. Bot.*, vol. 58, no. 3, pp. 259–267, Oct. 1997, doi:](http://paperpile.com/b/I9CICW/220l) [10.1016/S0304-3770(97)00039-9.](http://dx.doi.org/10.1016/S0304-3770(97)00039-9.)

[16] [A. Rattray, D. Ierodiaconou, J. Monk, V. L. Versace, and L. J. B. Laurenson, “Detecting patterns of change in benthic habitats by acoustic remote sensing,” *Mar. Ecol. Prog. Ser.*, vol. 477, pp. 1–13, 2013, [Online]. Available:](http://paperpile.com/b/I9CICW/OVFL) <https://www.int-res.com/abstracts/meps/v477/p1-13/>

[17] [M. Montefalcone, A. Rovere, V. Parravicini, G. Albertelli, C. Morri, and C. N. Bianchi, “Reprint of ‘Evaluating change in seagrass meadows: A time-framed comparison of Side Scan Sonar maps,’” *Aquat. Bot.*, vol. 115, pp. 36–44, Apr. 2014, doi:](http://paperpile.com/b/I9CICW/JANe) [10.1016/j.aquabot.2014.02.001.](http://dx.doi.org/10.1016/j.aquabot.2014.02.001.)

[18] [D. March, J. Alós, M. Cabanellas-Reboredo, E. Infantes, A. Jordi, and M. Palmer, “A Bayesian spatial approach for predicting seagrass occurrence,” *Estuar. Coast. Shelf Sci.*, vol. 131, pp. 206–212, Oct. 2013, doi:](http://paperpile.com/b/I9CICW/NF4k) [10.1016/j.ecss.2013.08.009.](http://dx.doi.org/10.1016/j.ecss.2013.08.009.)

[19] [C. A. Berlanga-Robles and A. Ruiz-Luna, “Assessing seasonal and long-term mangrove canopy variations in Sinaloa, northwest Mexico, based on time series of enhanced vegetation index (EVI) data,” *Wetlands Ecol. Manage.*, vol. 28, no. 2, pp. 229–249, Apr. 2020, doi:](http://paperpile.com/b/I9CICW/wpTt) [10.1007/s11273-020-09709-0.](http://dx.doi.org/10.1007/s11273-020-09709-0.)

[20] [C. L. Lopes, R. Mendes, I. Caçador, and J. M. Dias, “Assessing salt marsh extent and condition changes with 35 years of Landsat imagery: Tagus Estuary case study,” *Remote Sens. Environ.*, vol. 247, p. 111939, Sep. 2020, doi:](http://paperpile.com/b/I9CICW/MRj7) [10.1016/j.rse.2020.111939.](http://dx.doi.org/10.1016/j.rse.2020.111939.)

[21] [G. Casal, N. Sánchez-Carnero, E. Sánchez-Rodríguez, and J. Freire, “Remote sensing with SPOT-4 for mapping kelp forests in turbid waters on the south European Atlantic shelf,” *Estuar. Coast. Shelf Sci.*, vol. 91, no. 3, pp. 371–378, Feb. 2011, doi:](http://paperpile.com/b/I9CICW/HMkv) [10.1016/j.ecss.2010.10.024.](http://dx.doi.org/10.1016/j.ecss.2010.10.024.)

[22] [K. Gupta *et al.*, “An index for discrimination of mangroves from non-mangroves using LANDSAT 8 OLI imagery,” *MethodsX*, vol. 5, pp. 1129–1139, Sep. 2018, doi:](http://paperpile.com/b/I9CICW/MQ4i) [10.1016/j.mex.2018.09.011.](http://dx.doi.org/10.1016/j.mex.2018.09.011.)

[23] [C. Sun, S. Fagherazzi, and Y. Liu, “Classification mapping of salt marsh vegetation by flexible monthly NDVI time-series using Landsat imagery,” *Estuar. Coast. Shelf Sci.*, vol. 213, pp. 61–80, Nov. 2018, doi:](http://paperpile.com/b/I9CICW/WWaD) [10.1016/j.ecss.2018.08.007.](http://dx.doi.org/10.1016/j.ecss.2018.08.007.)

[24] [V. Otero, K. Quisthoudt, N. Koedam, and F. Dahdouh-Guebas, “Mangroves at Their Limits: Detection and Area Estimation of Mangroves along the Sahara Desert Coast,” *Remote Sensing*, vol. 8, no. 6, p. 512, Jun. 2016, doi:](http://paperpile.com/b/I9CICW/4Wbb) [10.3390/rs8060512.](http://dx.doi.org/10.3390/rs8060512.)

[25] [R. Baumstark, B. Dixon, P. Carlson, D. Palandro, and K. Kolasa, “Alternative spatially enhanced integrative techniques for mapping seagrass in Florida’s marine ecosystem,” *Int. J. Remote Sens.*, vol. 34, no. 4, pp. 1248–1264, Feb. 2013, doi:](http://paperpile.com/b/I9CICW/GLjT) [10.1080/01431161.2012.721941.](http://dx.doi.org/10.1080/01431161.2012.721941.)

[26] [J. A. Urbański, A. Mazur, and U. Janas, “Object-oriented classification of QuickBird data for mapping seagrass spatial structure,” *Oceanol. Hydrobiol. Stud.*, vol. 38, no. 1, pp. 27–43, 2009, [Online]. Available:](http://paperpile.com/b/I9CICW/mvC9) <https://www.academia.edu/download/50410875/Object-oriented_classification_of_QuickB20161118-22749-et6l2q.pdf>

[27] [R. Li, J.-K. Liu, A. Sukcharoenpong, J. Yuan, H. Zhu, and S. Zhang, “A Systematic Approach toward Detection of Seagrass Patches from Hyperspectral Imagery,” *Mar. Geod.*, vol. 35, no. 3, pp. 271–286, Jul. 2012, doi:](http://paperpile.com/b/I9CICW/buxp) [10.1080/01490419.2012.699019.](http://dx.doi.org/10.1080/01490419.2012.699019.)

[28] [A. L. Mitchell, R. M. Lucas, B. E. Donnelly, K. Pfitzner, A. K. Milne, and M. Finlayson, “A new map of mangroves for Kakadu National Park, Northern Australia, based on stereo aerial photography,” *Aquat. Conserv.*, vol. 17, no. 5, pp. 446–467, Jul. 2007, doi:](http://paperpile.com/b/I9CICW/R68l) [10.1002/aqc.818.](http://dx.doi.org/10.1002/aqc.818.)

[29] [V. Pasqualini, C. Pergent-Martini, P. Clabaut, and G. Pergent, “Mapping ofPosidonia oceanicausing Aerial Photographs and Side Scan Sonar: Application off the Island of Corsica (France),” *Estuar. Coast. Shelf Sci.*, vol. 47, no. 3, pp. 359–367, Sep. 1998, doi:](http://paperpile.com/b/I9CICW/yNgU) [10.1006/ecss.1998.0361.](http://dx.doi.org/10.1006/ecss.1998.0361.)

[30] [T. R. Marshall and P. F. Lee, “Mapping aquatic macrophytes through digital image analysis of aerial photographs: an assessment,” *J. Aquat. Plant Manag.*, vol. 32, no. 1, pp. 61–66, 1994, [Online]. Available:](http://paperpile.com/b/I9CICW/fuzx) <https://www.apms.org/wp-content/uploads/japm-32-02-061.pdf>

[31] [A. V. Uhrin and P. A. Townsend, “Improved seagrass mapping using linear spectral unmixing of aerial photographs,” *Estuar. Coast. Shelf Sci.*, vol. 171, pp. 11–22, Mar. 2016, doi:](http://paperpile.com/b/I9CICW/lbF0) [10.1016/j.ecss.2016.01.021.](http://dx.doi.org/10.1016/j.ecss.2016.01.021.)

[32] [A. J. Hsu, J. Kumagai, F. Favoretto, J. Dorian, B. Guerrero Martinez, and O. Aburto-Oropeza, “Driven by Drones: Improving Mangrove Extent Maps Using High-Resolution Remote Sensing,” *Remote Sensing*, vol. 12, no. 23, p. 3986, Dec. 2020, doi:](http://paperpile.com/b/I9CICW/7rfL) [10.3390/rs12233986.](http://dx.doi.org/10.3390/rs12233986.)

[33] [J. R. Krause, A. Hinojosa-Corona, A. B. Gray, and E. Burke Watson, “Emerging Sensor Platforms Allow for Seagrass Extent Mapping in a Turbid Estuary and from the Meadow to Ecosystem Scale,” *Remote Sensing*, vol. 13, no. 18, p. 3681, Sep. 2021, doi:](http://paperpile.com/b/I9CICW/PsZo) [10.3390/rs13183681.](http://dx.doi.org/10.3390/rs13183681.)

[34] [N. K. Nahirnick *et al.*, “Mapping with confidence; delineating seagrass habitats using Unoccupied Aerial Systems (UAS),” *Remote Sens. Ecol. Conserv.*, vol. 5, no. 2, pp. 121–135, Jun. 2019, doi:](http://paperpile.com/b/I9CICW/v4Qp) [10.1002/rse2.98.](http://dx.doi.org/10.1002/rse2.98.)

[35] [D. M. Price *et al.*, “Quantifying the Intra-Habitat Variation of Seagrass Beds with Unoccupied Aerial Vehicles (UAVs),” *Remote Sensing*, vol. 14, no. 3, p. 480, Jan. 2022, doi:](http://paperpile.com/b/I9CICW/UcVS) [10.3390/rs14030480.](http://dx.doi.org/10.3390/rs14030480.)

[36] [D. James, A. Collin, T. Houet, A. Mury, H. Gloria, and N. Le Poulain, “Towards Better Mapping of Seagrass Meadows using UAV Multispectral and Topographic Data,” *J. Coast. Res.*, vol. 95, no. SI, pp. 1117–1121, Jun. 2020, doi:](http://paperpile.com/b/I9CICW/BuKw) [10.2112/SI95-217.1.](http://dx.doi.org/10.2112/SI95-217.1.)

[37] [Y. Hu *et al.*, “Mapping coastal salt marshes in China using time series of Sentinel-1 SAR,” *ISPRS J. Photogramm. Remote Sens.*, vol. 173, pp. 122–134, Mar. 2021, doi:](http://paperpile.com/b/I9CICW/D8kZ) [10.1016/j.isprsjprs.2021.01.003.](http://dx.doi.org/10.1016/j.isprsjprs.2021.01.003.)

[38] [G. Ashiagbor, W. A. Asante, J. A. Quaye-Ballard, E. K. Forkuo, E. Acheampong, and E. Foli, “Mangrove mapping using Sentinel-1 data for improved decision support on sustainable conservation and restoration interventions in the Keta Lagoon Complex Ramsar Site, Ghana,” *Mar. Freshwater Res.*, vol. 72, no. 11, pp. 1588–1601, 2021, [Online]. Available:](http://paperpile.com/b/I9CICW/lrjG) <https://www.publish.csiro.au/MF/MF20105>

[39] [A. Abdel-Hamid, O. Dubovyk, I. Abou El-Magd, and G. Menz, “Mapping Mangroves Extents on the Red Sea Coastline in Egypt using Polarimetric SAR and High Resolution Optical Remote Sensing Data,” *Sustain. Sci. Pract. Policy*, vol. 10, no. 3, p. 646, Feb. 2018, doi:](http://paperpile.com/b/I9CICW/ZzUU) [10.3390/su10030646.](http://dx.doi.org/10.3390/su10030646.)

[40] [M. Simard, G. D. Grandi, S. Saatchi, and P. Mayaux, “Mapping tropical coastal vegetation using JERS-1 and ERS-1 radar data with a decision tree classifier,” *Int. J. Remote Sens.*, vol. 23, no. 7, pp. 1461–1474, Jan. 2002, doi:](http://paperpile.com/b/I9CICW/P6Ld) [10.1080/01431160110092984.](http://dx.doi.org/10.1080/01431160110092984.)

[41] [L. San Martín, N. S. Morandeira, R. Grimson, M. Rajngewerc, E. B. González, and P. Kandus, “The contribution of ALOS/PALSAR-1 multi-temporal data to map permanently and temporarily flooded coastal wetlands,” *Int. J. Remote Sens.*, vol. 41, no. 4, pp. 1582–1602, Feb. 2020, doi:](http://paperpile.com/b/I9CICW/yBbJ) [10.1080/01431161.2019.1673915.](http://dx.doi.org/10.1080/01431161.2019.1673915.)

[42] [L. B. van Ardenne and G. L. Chmura, “Applying Airborne LiDAR to Map Salt Marsh Inland Boundaries,” *Remote Sensing*, vol. 13, no. 21, p. 4245, Oct. 2021, doi:](http://paperpile.com/b/I9CICW/yBsz) [10.3390/rs13214245.](http://dx.doi.org/10.3390/rs13214245.)

[43] [G. Chust, M. Grande, I. Galparsoro, A. Uriarte, and Á. Borja, “Capabilities of the bathymetric Hawk Eye LiDAR for coastal habitat mapping: A case study within a Basque estuary,” *Estuar. Coast. Shelf Sci.*, vol. 89, no. 3, pp. 200–213, Oct. 2010, doi:](http://paperpile.com/b/I9CICW/zyAa) [10.1016/j.ecss.2010.07.002.](http://dx.doi.org/10.1016/j.ecss.2010.07.002.)

[44] [A. Micallef, T. P. Le Bas, V. A. I. Huvenne, P. Blondel, V. Hühnerbach, and A. Deidun, “A multi-method approach for benthic habitat mapping of shallow coastal areas with high-resolution multibeam data,” *Cont. Shelf Res.*, vol. 39–40, pp. 14–26, May 2012, doi:](http://paperpile.com/b/I9CICW/eS0K) [10.1016/j.csr.2012.03.008.](http://dx.doi.org/10.1016/j.csr.2012.03.008.)

[45] [M. Montefalcone, G. Albertelli, C. Nike Bianchi, M. Mariani, and C. Morri, “A new synthetic index and a protocol for monitoring the status ofPosidonia oceanica meadows: a case study at Sanremo (Ligurian Sea, NW Mediterranean),” *Aquat. Conserv.*, vol. 16, no. 1, pp. 29–42, Jan. 2006, doi:](http://paperpile.com/b/I9CICW/o0Zs) [10.1002/aqc.688.](http://dx.doi.org/10.1002/aqc.688.)

[46] [A. Greene, A. F. Rahman, R. Kline, and M. S. Rahman, “Side scan sonar: A cost-efficient alternative method for measuring seagrass cover in shallow environments,” *Estuar. Coast. Shelf Sci.*, vol. 207, pp. 250–258, Jul. 2018, doi:](http://paperpile.com/b/I9CICW/oxwZ) [10.1016/j.ecss.2018.04.017.](http://dx.doi.org/10.1016/j.ecss.2018.04.017.)

[47] [A. Kruss, J. Tęgowski, A. Tatarek, J. Wiktor, and P. Blondel, “Spatial distribution of macroalgae along the shores of Kongsfjorden (West Spitsbergen) using acoustic imaging,” *Pol. Polar Res.*, vol. 38, no. 2, pp. 205–229, Jun. 2017, doi:](http://paperpile.com/b/I9CICW/iRoR) [10.1515/popore-2017-0009.](http://dx.doi.org/10.1515/popore-2017-0009.)

[48] [J. Gu *et al.*, “Areal Extent, Species Composition, and Spatial Distribution of Coastal Saltmarshes in China,” *IEEE Journal of Selected Topics in Applied Earth Observations and Remote Sensing*, vol. 14, pp. 7085–7094, 2021, doi:](http://paperpile.com/b/I9CICW/tZqy) [10.1109/JSTARS.2021.3093673.](http://dx.doi.org/10.1109/JSTARS.2021.3093673.)

[49] [A. Davranche, G. Lefebvre, and B. Poulin, “Wetland monitoring using classification trees and SPOT-5 seasonal time series,” *Remote Sens. Environ.*, vol. 114, no. 3, pp. 552–562, Mar. 2010, doi:](http://paperpile.com/b/I9CICW/3Nne) [10.1016/j.rse.2009.10.009.](http://dx.doi.org/10.1016/j.rse.2009.10.009.)

[50] [M. Wang *et al.*, “Assessing Texture Features to Classify Coastal Wetland Vegetation from High Spatial Resolution Imagery Using Completed Local Binary Patterns (CLBP),” *Remote Sensing*, vol. 10, no. 5, p. 778, May 2018, doi:](http://paperpile.com/b/I9CICW/0idS) [10.3390/rs10050778.](http://dx.doi.org/10.3390/rs10050778.)

[51] [M. Lyons, S. Phinn, and C. Roelfsema, “Integrating Quickbird Multi-Spectral Satellite and Field Data: Mapping Bathymetry, Seagrass Cover, Seagrass Species and Change in Moreton Bay, Australia in 2004 and 2007,” *Remote Sensing*, vol. 3, no. 1, pp. 42–64, Jan. 2011, doi:](http://paperpile.com/b/I9CICW/j76y) [10.3390/rs3010042.](http://dx.doi.org/10.3390/rs3010042.)

[52] [S. Chakravortty, J. Li, and A. Plaza, “A Technique for Subpixel Analysis of Dynamic Mangrove Ecosystems With Time-Series Hyperspectral Image Data,” *IEEE Journal of Selected Topics in Applied Earth Observations and Remote Sensing*, vol. 11, no. 4, pp. 1244–1252, Apr. 2018, doi:](http://paperpile.com/b/I9CICW/PTLp) [10.1109/JSTARS.2017.2782324.](http://dx.doi.org/10.1109/JSTARS.2017.2782324.)

[53] [T. Kumar, S. Panigrahy, P. Kumar, and J. S. Parihar, “Classification of floristic composition of mangrove forests using hyperspectral data: case study of Bhitarkanika National Park, India,” *J. Coast. Conserv.*, vol. 17, no. 1, pp. 121–132, Mar. 2013, doi:](http://paperpile.com/b/I9CICW/2LCE) [10.1007/s11852-012-0223-2.](http://dx.doi.org/10.1007/s11852-012-0223-2.)

[54] [A. J. Meehan, R. J. Williams, and F. A. Watford, “Detecting trends in seagrass abundance using aerial photograph interpretation: Problems arising with the evolution of mapping methods,” *Estuaries*, vol. 28, no. 3, pp. 462–472, Jun. 2005, doi:](http://paperpile.com/b/I9CICW/jnNH) [10.1007/BF02693927.](http://dx.doi.org/10.1007/BF02693927.)

[55] [C. B. Higinbotham, M. Alber, and A. G. Chalmers, “Analysis of tidal marsh vegetation patterns in two Georgia estuaries using aerial photography and GIS,” *Estuaries*, vol. 27, no. 4, pp. 670–683, Aug. 2004, doi:](http://paperpile.com/b/I9CICW/8lSM) [10.1007/BF02907652.](http://dx.doi.org/10.1007/BF02907652.)

[56] [L. Li, S. L. Ustin, and M. Lay, “Application of multiple endmember spectral mixture analysis (MESMA) to AVIRIS imagery for coastal salt marsh mapping: a case study in China Camp, CA, USA,” *Int. J. Remote Sens.*, vol. 26, no. 23, pp. 5193–5207, Dec. 2005, doi:](http://paperpile.com/b/I9CICW/xu3f) [10.1080/01431160500218911.](http://dx.doi.org/10.1080/01431160500218911.)

[57] [T. Kumar, K. Preeti, K. Chandrasekar, and S. Bandyopadhyay, “Aviris–ng hyperspectral data for mapping mangrove forests and their health spatially: A case study of Indian Sundarbans,” *J. Trop. For. Sci.*, vol. 32, no. 3, pp. 317–331, Aug. 2020, doi:](http://paperpile.com/b/I9CICW/ftEM) [10.26525/jtfs2020.32.3.317.](http://dx.doi.org/10.26525/jtfs2020.32.3.317.)

[58] [L. Tait, J. Bind, H. Charan-Dixon, I. Hawes, J. Pirker, and D. Schiel, “Unmanned Aerial Vehicles (UAVs) for Monitoring Macroalgal Biodiversity: Comparison of RGB and Multispectral Imaging Sensors for Biodiversity Assessments,” *Remote Sensing*, vol. 11, no. 19, p. 2332, Oct. 2019, doi:](http://paperpile.com/b/I9CICW/qNZc) [10.3390/rs11192332.](http://dx.doi.org/10.3390/rs11192332.)

[59] [A. C. G. Schimel, C. J. Brown, and D. Ierodiaconou, “Automated Filtering of Multibeam Water-Column Data to Detect Relative Abundance of Giant Kelp (Macrocystis pyrifera),” *Remote Sensing*, vol. 12, no. 9, p. 1371, Apr. 2020, doi:](http://paperpile.com/b/I9CICW/ZFCQ) [10.3390/rs12091371.](http://dx.doi.org/10.3390/rs12091371.)

[60] [S. Sonoki *et al.*, “Using Acoustics to Determine Eelgrass Bed Distribution and to Assess the Seasonal Variation of Ecosystem Service,” *PLoS One*, vol. 11, no. 3, p. e0150890, Mar. 2016, doi:](http://paperpile.com/b/I9CICW/Vf8C) [10.1371/journal.pone.0150890.](http://dx.doi.org/10.1371/journal.pone.0150890.)

[61] [S. Benmokhtar, M. Robin, M. Maanan, and H. Bazairi, “Mapping and Quantification of the Dwarf Eelgrass Zostera noltei Using a Random Forest Algorithm on a SPOT 7 Satellite Image,” *ISPRS International Journal of Geo-Information*, vol. 10, no. 5, p. 313, May 2021, doi:](http://paperpile.com/b/I9CICW/LD2Q) [10.3390/ijgi10050313.](http://dx.doi.org/10.3390/ijgi10050313.)

[62] [D. Wang, B. Wan, P. Qiu, Y. Su, Q. Guo, and X. Wu, “Artificial Mangrove Species Mapping Using Pléiades-1: An Evaluation of Pixel-Based and Object-Based Classifications with Selected Machine Learning Algorithms,” *Remote Sensing*, vol. 10, no. 2, p. 294, Feb. 2018, doi:](http://paperpile.com/b/I9CICW/qhCf) [10.3390/rs10020294.](http://dx.doi.org/10.3390/rs10020294.)

[63] [L. Peng, K. Liu, J. Cao, Y. Zhu, F. Li, and L. Liu, “Combining GF-2 and RapidEye satellite data for mapping mangrove species using ensemble machine-learning methods,” *Int. J. Remote Sens.*, vol. 41, no. 3, pp. 813–838, Feb. 2020, doi:](http://paperpile.com/b/I9CICW/Sbp5) [10.1080/01431161.2019.1648907.](http://dx.doi.org/10.1080/01431161.2019.1648907.)

[64] [X. Huang, L. Zhang, and L. Wang, “Evaluation of Morphological Texture Features for Mangrove Forest Mapping and Species Discrimination Using Multispectral IKONOS Imagery,” *IEEE Geoscience and Remote Sensing Letters*, vol. 6, no. 3, pp. 393–397, Jul. 2009, doi:](http://paperpile.com/b/I9CICW/JSp0) [10.1109/LGRS.2009.2014398.](http://dx.doi.org/10.1109/LGRS.2009.2014398.)

[65] [S. Chakravortty, “Analysis of end member detection and subpixel classification algorithms on hyperspectral imagery for tropical mangrove species discrimination in the Sunderbans Delta, India,” *Journal of Applied Remote Sensing*, vol. 7, no. 1. p. 073523, 2013. doi:](http://paperpile.com/b/I9CICW/YwH5) [10.1117/1.jrs.7.073523.](http://dx.doi.org/10.1117/1.jrs.7.073523.)

[66] [S. Chakravortty and S. Chakrabarti, “Design and Development of Higher Order Spectral Unmixing Model for Mangrove Species Discrimination,” *Proc. Nat. Acad. Sci. India Sect. A*, vol. 87, no. 4, pp. 557–566, Dec. 2017, doi:](http://paperpile.com/b/I9CICW/AcJ5) [10.1007/s40010-017-0434-x.](http://dx.doi.org/10.1007/s40010-017-0434-x.)

[67] [W. Koedsin and C. Vaiphasa, “Discrimination of Tropical Mangroves at the Species Level with EO-1 Hyperion Data,” *Remote Sensing*, vol. 5, no. 7, pp. 3562–3582, Jul. 2013, doi:](http://paperpile.com/b/I9CICW/RYBx) [10.3390/rs5073562.](http://dx.doi.org/10.3390/rs5073562.)

[68] [M. K. Heenkenda, K. E. Joyce, S. W. Maier, and R. Bartolo, “Mangrove Species Identification: Comparing WorldView-2 with Aerial Photographs,” *Remote Sensing*, vol. 6, no. 7, pp. 6064–6088, Jun. 2014, doi:](http://paperpile.com/b/I9CICW/rR4D) [10.3390/rs6076064.](http://dx.doi.org/10.3390/rs6076064.)

[69] [G. Casal, T. Kutser, J. A. Domínguez-Gómez, N. Sánchez-Carnero, and J. Freire, “Assessment of the hyperspectral sensor CASI-2 for macroalgal discrimination on the Ría de Vigo coast (NW Spain) using field spectroscopy and modelled spectral libraries,” *Continental Shelf Research*, vol. 55. pp. 129–140, 2013. doi:](http://paperpile.com/b/I9CICW/fIbh) [10.1016/j.csr.2013.01.010.](http://dx.doi.org/10.1016/j.csr.2013.01.010.)

[70] [F. J. Artigas and J. S. Yang, “Hyperspectral remote sensing of marsh species and plant vigour gradient in the New Jersey Meadowlands,” *Int. J. Remote Sens.*, vol. 26, no. 23, pp. 5209–5220, Dec. 2005, doi:](http://paperpile.com/b/I9CICW/HP7s) [10.1080/01431160500218952.](http://dx.doi.org/10.1080/01431160500218952.)

[71] [M. Valle *et al.*, “Mapping estuarine habitats using airborne hyperspectral imagery, with special focus on seagrass meadows,” *Estuar. Coast. Shelf Sci.*, vol. 164, pp. 433–442, Oct. 2015, doi:](http://paperpile.com/b/I9CICW/vIrj) [10.1016/j.ecss.2015.07.034.](http://dx.doi.org/10.1016/j.ecss.2015.07.034.)

[72] [S.-I. Park, Y.-S. Hwang, and J.-S. Um, “Estimating blue carbon accumulated in a halophyte community using UAV imagery: a case study of the southern coastal wetlands in South Korea,” *J. Coast. Conserv.*, vol. 25, no. 3, p. 38, May 2021, doi:](http://paperpile.com/b/I9CICW/dzZw) [10.1007/s11852-021-00828-1.](http://dx.doi.org/10.1007/s11852-021-00828-1.)

[73] [J. Cao, W. Leng, K. Liu, L. Liu, Z. He, and Y. Zhu, “Object-Based Mangrove Species Classification Using Unmanned Aerial Vehicle Hyperspectral Images and Digital Surface Models,” *Remote Sensing*, vol. 10, no. 1, p. 89, Jan. 2018, doi:](http://paperpile.com/b/I9CICW/PSYF) [10.3390/rs10010089.](http://dx.doi.org/10.3390/rs10010089.)

[74] [N. K. Nahirnick, P. Hunter, M. Costa, S. Schroeder, and T. Sharma, “Benefits and Challenges of UAS Imagery for Eelgrass (Zostera marina) Mapping in Small Estuaries of the Canadian West Coast,” *J. Coast. Res.*, vol. 35, no. 3, pp. 673–683, Feb. 2019, doi:](http://paperpile.com/b/I9CICW/Cwak) [10.2112/JCOASTRES-D-18-00079.1.](http://dx.doi.org/10.2112/JCOASTRES-D-18-00079.1.)

[75] [J. Cao, K. Liu, L. Zhuo, L. Liu, Y. Zhu, and L. Peng, “Combining UAV-based hyperspectral and LiDAR data for mangrove species classification using the rotation forest algorithm,” *Int. J. Appl. Earth Obs. Geoinf.*, vol. 102, p. 102414, Oct. 2021, doi:](http://paperpile.com/b/I9CICW/VCH5) [10.1016/j.jag.2021.102414.](http://dx.doi.org/10.1016/j.jag.2021.102414.)

[76] [J. A. A. Castillo, A. A. Apan, T. N. Maraseni, and S. G. Salmo, “Estimation and mapping of above-ground biomass of mangrove forests and their replacement land uses in the Philippines using Sentinel imagery,” *ISPRS J. Photogramm. Remote Sens.*, vol. 134, pp. 70–85, Dec. 2017, doi:](http://paperpile.com/b/I9CICW/VWPa) [10.1016/j.isprsjprs.2017.10.016.](http://dx.doi.org/10.1016/j.isprsjprs.2017.10.016.)

[77] [H.-H. Nguyen, H. D. Vu, and A. Röder, “Estimation of above-ground mangrove biomass using Landsat-8 data- derived vegetation indices: A case study in Quang Ninh Province, Vietnam,” *For. Soc.*, pp. 506–525, Oct. 2021, doi:](http://paperpile.com/b/I9CICW/7FWq) [10.24259/fs.v5i2.13755.](http://dx.doi.org/10.24259/fs.v5i2.13755.)

[78] [M. G. T. Portela, G. M. de Espindola, G. S. Valladares, J. V. A. Amorim, and J. C. O. Frota, “Vegetation biomass and carbon stocks in the Parnaíba River Delta, NE Brazil,” *Wetlands Ecol. Manage.*, vol. 28, no. 4, pp. 607–622, Aug. 2020, doi:](http://paperpile.com/b/I9CICW/tLqx) [10.1007/s11273-020-09735-y.](http://dx.doi.org/10.1007/s11273-020-09735-y.)

[79] [A. Knudby and L. Nordlund, “Remote sensing of seagrasses in a patchy multi-species environment,” *Int. J. Remote Sens.*, vol. 32, no. 8, pp. 2227–2244, Mar. 2011, doi:](http://paperpile.com/b/I9CICW/91js) [10.1080/01431161003692057.](http://dx.doi.org/10.1080/01431161003692057.)

[80] [Y. Hirata, R. Tabuchi, P. Patanaponpaiboon, S. Poungparn, R. Yoneda, and Y. Fujioka, “Estimation of aboveground biomass in mangrove forests using high-resolution satellite data,” *J. Forest Res.*, vol. 19, no. 1, pp. 34–41, Feb. 2014, doi:](http://paperpile.com/b/I9CICW/x57E) [10.1007/s10310-013-0402-5.](http://dx.doi.org/10.1007/s10310-013-0402-5.)

[81] [A. Anand *et al.*, “Use of Hyperion for Mangrove Forest Carbon Stock Assessment in Bhitarkanika Forest Reserve: A Contribution Towards Blue Carbon Initiative,” *Remote Sensing*, vol. 12, no. 4, p. 597, Feb. 2020, doi:](http://paperpile.com/b/I9CICW/Djiy) [10.3390/rs12040597.](http://dx.doi.org/10.3390/rs12040597.)

[82] [A. R. Jones, R. Raja Segaran, K. D. Clarke, M. Waycott, W. S. H. Goh, and B. M. Gillanders, “Estimating Mangrove Tree Biomass and Carbon Content: A Comparison of Forest Inventory Techniques and Drone Imagery,” *Frontiers in Marine Science*, vol. 6, 2020, doi:](http://paperpile.com/b/I9CICW/QNOh) [10.3389/fmars.2019.00784.](http://dx.doi.org/10.3389/fmars.2019.00784.)

[83] [A. Wirasatriya *et al.*, “Mangrove Above-Ground Biomass and Carbon Stock in the Karimunjawa-Kemujan Islands Estimated from Unmanned Aerial Vehicle-Imagery,” *Sustain. Sci. Pract. Policy*, vol. 14, no. 2, p. 706, Jan. 2022, doi:](http://paperpile.com/b/I9CICW/VxVa) [10.3390/su14020706.](http://dx.doi.org/10.3390/su14020706.)

[84] [C. L. Doughty and K. C. Cavanaugh, “Mapping Coastal Wetland Biomass from High Resolution Unmanned Aerial Vehicle (UAV) Imagery,” *Remote Sensing*, vol. 11, no. 5, p. 540, Mar. 2019, doi:](http://paperpile.com/b/I9CICW/Gt1i) [10.3390/rs11050540.](http://dx.doi.org/10.3390/rs11050540.)

[85] [M. K. Nesha, Y. A. Hussin, L. M. van Leeuwen, and Y. B. Sulistioadi, “Modeling and mapping aboveground biomass of the restored mangroves using ALOS-2 PALSAR-2 in East Kalimantan, Indonesia,” *Int. J. Appl. Earth Obs. Geoinf.*, vol. 91, p. 102158, Sep. 2020, doi:](http://paperpile.com/b/I9CICW/a9KX) [10.1016/j.jag.2020.102158.](http://dx.doi.org/10.1016/j.jag.2020.102158.)

[86] [V. N. Luong, T. T. Tu, A. L. Khoi, X. T. Hong, T. N. Hoan, and T. L. H. Thuy, “Biomass estimation and mapping of can Gio mangrove biosphere reserve in south of Viet Nam using Alos-2 palsar-2 data,” *Appl. Ecol. Environ. Res.*, vol. 17, no. 1, pp. 15–31, 2019, doi:](http://paperpile.com/b/I9CICW/gYrl) [10.15666/aeer/1701_015031.](http://dx.doi.org/10.15666/aeer/1701_015031.)

[87] [T. D. Pham *et al.*, “Estimating Mangrove Above-Ground Biomass Using Extreme Gradient Boosting Decision Trees Algorithm with Fused Sentinel-2 and ALOS-2 PALSAR-2 Data in Can Gio Biosphere Reserve, Vietnam,” *Remote Sensing*, vol. 12, no. 5. p. 777, 2020. doi:](http://paperpile.com/b/I9CICW/ZWQD) [10.3390/rs12050777.](http://dx.doi.org/10.3390/rs12050777.)

[88] [O. Hamdan, H. Khali Aziz, and I. Mohd Hasmadi, “L-band ALOS PALSAR for biomass estimation of Matang Mangroves, Malaysia,” *Remote Sens. Environ.*, vol. 155, pp. 69–78, Dec. 2014, doi:](http://paperpile.com/b/I9CICW/kWlv) [10.1016/j.rse.2014.04.029.](http://dx.doi.org/10.1016/j.rse.2014.04.029.)

[89] [X. Li *et al.*, “Regression and analytical models for estimating mangrove wetland biomass in South China using Radarsat images,” *Int. J. Remote Sens.*, vol. 28, no. 24, pp. 5567–5582, Dec. 2007, doi:](http://paperpile.com/b/I9CICW/M6BB) [10.1080/01431160701227638.](http://dx.doi.org/10.1080/01431160701227638.)

[90] [Y. Tian *et al.*, “Aboveground mangrove biomass estimation in Beibu Gulf using machine learning and UAV remote sensing,” *Sci. Total Environ.*, vol. 781, p. 146816, Aug. 2021, doi:](http://paperpile.com/b/I9CICW/V4hr) [10.1016/j.scitotenv.2021.146816.](http://dx.doi.org/10.1016/j.scitotenv.2021.146816.)

[91] [S. M. Ghosh, M. D. Behera, and S. Paramanik, “Canopy Height Estimation Using Sentinel Series Images through Machine Learning Models in a Mangrove Forest,” *Remote Sensing*, vol. 12, no. 9, p. 1519, May 2020, doi:](http://paperpile.com/b/I9CICW/qcse) [10.3390/rs12091519.](http://dx.doi.org/10.3390/rs12091519.)

[92] [A. Yaney-Keller, P. Santidrián Tomillo, J. M. Marshall, and F. V. Paladino, “Using Unmanned Aerial Systems (UAS) to assay mangrove estuaries on the Pacific coast of Costa Rica,” *PLoS One*, vol. 14, no. 6, p. e0217310, Jun. 2019, doi:](http://paperpile.com/b/I9CICW/mZ0I) [10.1371/journal.pone.0217310.](http://dx.doi.org/10.1371/journal.pone.0217310.)

[93] [C. C. Trettin *et al.*, “Mangrove carbon stocks in Pongara National Park, Gabon,” *Estuar. Coast. Shelf Sci.*, vol. 259, p. 107432, Sep. 2021, doi:](http://paperpile.com/b/I9CICW/9Tar) [10.1016/j.ecss.2021.107432.](http://dx.doi.org/10.1016/j.ecss.2021.107432.)

[94] [A. Aslan, A. F. Rahman, and S. M. Robeson, “Investigating the use of Alos Prism data in detecting mangrove succession through canopy height estimation,” *Ecol. Indic.*, vol. 87, pp. 136–143, Apr. 2018, doi:](http://paperpile.com/b/I9CICW/wDA2) [10.1016/j.ecolind.2017.12.008.](http://dx.doi.org/10.1016/j.ecolind.2017.12.008.)

[95] [J. M. Kovacs, C. V. Vandenberg, J. Wang, and F. Flores-Verdugo, “The Use of Multipolarized Spaceborne SAR Backscatter for Monitoring the Health of a Degraded Mangrove Forest,” *J. Coast. Res.*, vol. 24, no. 1 (241), pp. 248–254, Jan. 2008, doi:](http://paperpile.com/b/I9CICW/YinI) [10.2112/06-0660.1.](http://dx.doi.org/10.2112/06-0660.1.)

[96] [E. A. Feliciano, S. Wdowinski, M. D. Potts, S.-K. Lee, and T. E. Fatoyinbo, “Estimating Mangrove Canopy Height and Above-Ground Biomass in the Everglades National Park with Airborne LiDAR and TanDEM-X Data,” *Remote Sensing*, vol. 9, no. 7, p. 702, Jul. 2017, doi:](http://paperpile.com/b/I9CICW/ts2T) [10.3390/rs9070702.](http://dx.doi.org/10.3390/rs9070702.)

[97] [R. B. Salum, S. A. Robinson, and K. Rogers, “A Validated and Accurate Method for Quantifying and Extrapolating Mangrove Above-Ground Biomass Using LiDAR Data,” *Remote Sensing*, vol. 13, no. 14, p. 2763, Jul. 2021, doi:](http://paperpile.com/b/I9CICW/WPGZ) [10.3390/rs13142763.](http://dx.doi.org/10.3390/rs13142763.)

[98] [W. Wannasiri, M. Nagai, K. Honda, P. Santitamnont, and P. Miphokasap, “Extraction of Mangrove Biophysical Parameters Using Airborne LiDAR,” *Remote Sensing*, vol. 5, no. 4, pp. 1787–1808, Apr. 2013, doi:](http://paperpile.com/b/I9CICW/L6kW) [10.3390/rs5041787.](http://dx.doi.org/10.3390/rs5041787.)

[99] [D. Yin and L. Wang, “Individual mangrove tree measurement using UAV-based LiDAR data: Possibilities and challenges,” *Remote Sens. Environ.*, vol. 223, pp. 34–49, Mar. 2019, doi:](http://paperpile.com/b/I9CICW/6p0z) [10.1016/j.rse.2018.12.034.](http://dx.doi.org/10.1016/j.rse.2018.12.034.)

[100] [M. Paul, A. Lefebvre, E. Manca, and C. L. Amos, “An acoustic method for the remote measurement of seagrass metrics,” *Estuar. Coast. Shelf Sci.*, vol. 93, no. 1, pp. 68–79, May 2011, doi:](http://paperpile.com/b/I9CICW/ohay) [10.1016/j.ecss.2011.04.006.](http://dx.doi.org/10.1016/j.ecss.2011.04.006.)

[101] [H. A.-E. Monsef and S. E. Smith, “A new approach for estimating mangrove canopy cover using Landsat 8 imagery,” *Comput. Electron. Agric.*, vol. 135, pp. 183–194, Apr. 2017, doi:](http://paperpile.com/b/I9CICW/GHv7) [10.1016/j.compag.2017.02.007.](http://dx.doi.org/10.1016/j.compag.2017.02.007.)

[102] [M. Ji and J. Feng, “Subpixel measurement of mangrove canopy closure via spectral mixture analysis,” *Front. Earth Sci.*, vol. 5, no. 2, pp. 130–137, Jun. 2011, doi:](http://paperpile.com/b/I9CICW/5x3U) [10.1007/s11707-011-0156-3.](http://dx.doi.org/10.1007/s11707-011-0156-3.)

[103] [G. Lassalle, M. P. Ferreira, L. E. C. La Rosa, and C. R. de Souza Filho, “Deep learning-based individual tree crown delineation in mangrove forests using very-high-resolution satellite imagery,” *ISPRS J. Photogramm. Remote Sens.*, vol. 189, pp. 220–235, Jul. 2022, doi:](http://paperpile.com/b/I9CICW/4rzf) [10.1016/j.isprsjprs.2022.05.002.](http://dx.doi.org/10.1016/j.isprsjprs.2022.05.002.)

[104] [M. K. Heenkenda, K. E. Joyce, and S. W. Maier, “Mangrove Tree Crown Delineation from High-Resolution Imagery,” *Photogrammetric Engineering & Remote Sensing*, vol. 81, no. 6, pp. 471–479, 2015, doi:](http://paperpile.com/b/I9CICW/L2hY) [10.14358/PERS.81.6.471.](http://dx.doi.org/10.14358/PERS.81.6.471.)

[105] [H. Biswas, K. Zhang, M. S. Ross, and D. Gann, “Delineation of Tree Patches in a Mangrove-Marsh Transition Zone by Watershed Segmentation of Aerial Photographs,” *Remote Sensing*, vol. 12, no. 13, p. 2086, Jun. 2020, doi:](http://paperpile.com/b/I9CICW/Rr8t) [10.3390/rs12132086.](http://dx.doi.org/10.3390/rs12132086.)

[106] [K. C. Cavanaugh, K. C. Cavanaugh, T. W. Bell, and E. G. Hockridge, “An Automated Method for Mapping Giant Kelp Canopy Dynamics from UAV,” *Front. Environ. Sci. Eng. China*, vol. 8, 2021, doi:](http://paperpile.com/b/I9CICW/UTyx) [10.3389/fenvs.2020.587354.](http://dx.doi.org/10.3389/fenvs.2020.587354.)

[107] [B. L. Edwards, S. T. Allen, D. H. Braud, and R. F. Keim, “Stand density and carbon storage in cypress-tupelo wetland forests of the Mississippi River delta,” *For. Ecol. Manage.*, vol. 441, pp. 106–114, Jun. 2019, doi:](http://paperpile.com/b/I9CICW/a2Rx) [10.1016/j.foreco.2019.03.046.](http://dx.doi.org/10.1016/j.foreco.2019.03.046.)

[108] [J. M. Kovacs, X. X. Lu, F. Flores-Verdugo, C. Zhang, F. Flores de Santiago, and X. Jiao, “Applications of ALOS PALSAR for monitoring biophysical parameters of a degraded black mangrove (Avicennia germinans) forest,” *ISPRS J. Photogramm. Remote Sens.*, vol. 82, pp. 102–111, Aug. 2013, doi:](http://paperpile.com/b/I9CICW/QwDg) [10.1016/j.isprsjprs.2013.05.004.](http://dx.doi.org/10.1016/j.isprsjprs.2013.05.004.)

[109] [J. M. Kovacs, X. Jiao, F. Flores-de-Santiago, C. Zhang, and F. Flores-Verdugo, “Assessing relationships between Radarsat-2 C-band and structural parameters of a degraded mangrove forest,” *Int. J. Remote Sens.*, vol. 34, no. 20, pp. 7002–7019, Oct. 2013, doi:](http://paperpile.com/b/I9CICW/zp48) [10.1080/01431161.2013.813090.](http://dx.doi.org/10.1080/01431161.2013.813090.)

[110] [K. D. Kanniah, C. S. Kang, S. Sharma, and A. A. Amir, “Remote Sensing to Study Mangrove Fragmentation and Its Impacts on Leaf Area Index and Gross Primary Productivity in the South of Peninsular Malaysia,” *Remote Sensing*, vol. 13, no. 8, p. 1427, Apr. 2021, doi:](http://paperpile.com/b/I9CICW/oBf5) [10.3390/rs13081427.](http://dx.doi.org/10.3390/rs13081427.)

[111] [S. Manna and B. Raychaudhuri, “Retrieval of Leaf area index and stress conditions for Sundarban mangroves using Sentinel-2 data,” *Int. J. Remote Sens.*, vol. 41, no. 3, pp. 1019–1039, Feb. 2020, doi:](http://paperpile.com/b/I9CICW/EIix) [10.1080/01431161.2019.1655174.](http://dx.doi.org/10.1080/01431161.2019.1655174.)

[112] [J. M. Kovacs, F. Flores-Verdugo, J. Wang, and L. P. Aspden, “Estimating leaf area index of a degraded mangrove forest using high spatial resolution satellite data,” *Aquat. Bot.*, vol. 80, no. 1, pp. 13–22, Sep. 2004, doi:](http://paperpile.com/b/I9CICW/F5jt) [10.1016/j.aquabot.2004.06.001.](http://dx.doi.org/10.1016/j.aquabot.2004.06.001.)

[113] [J. M. Kovacs, J. M. L. King, F. Flores de Santiago, and F. Flores-Verdugo, “Evaluating the condition of a mangrove forest of the Mexican Pacific based on an estimated leaf area index mapping approach,” *Environ. Monit. Assess.*, vol. 157, no. 1–4, pp. 137–149, Oct. 2009, doi:](http://paperpile.com/b/I9CICW/vdDk) [10.1007/s10661-008-0523-z.](http://dx.doi.org/10.1007/s10661-008-0523-z.)

[114] [R. George, H. Padalia, S. K. Sinha, and A. S. Kumar, “Evaluation of the Use of Hyperspectral Vegetation Indices for Estimating Mangrove Leaf Area Index in Middle Andaman Island, India,” *Remote Sens. Lett.*, vol. 9, no. 11, pp. 1099–1108, Nov. 2018, doi:](http://paperpile.com/b/I9CICW/PlVB) [10.1080/2150704X.2018.1508910.](http://dx.doi.org/10.1080/2150704X.2018.1508910.)

[115] [X. Liu and L. Wang, “Feasibility of using consumer-grade unmanned aerial vehicles to estimate leaf area index in Mangrove forest,” *Remote Sens. Lett.*, vol. 9, no. 11, pp. 1040–1049, Nov. 2018, doi:](http://paperpile.com/b/I9CICW/sIvq) [10.1080/2150704X.2018.1504339.](http://dx.doi.org/10.1080/2150704X.2018.1504339.)

[116] [S. Ghosh, D. R. Mishra, and A. A. Gitelson, “Long-term monitoring of biophysical characteristics of tidal wetlands in the northern Gulf of Mexico — A methodological approach using MODIS,” *Remote Sens. Environ.*, vol. 173, pp. 39–58, Feb. 2016, doi:](http://paperpile.com/b/I9CICW/VlWf) [10.1016/j.rse.2015.11.015.](http://dx.doi.org/10.1016/j.rse.2015.11.015.)

[117] [B. R. Parida and A. Kumari, “Mapping and modeling mangrove biophysical and biochemical parameters using Sentinel-2A satellite data in Bhitarkanika National Park, Odisha,” *Modeling Earth Systems and Environment*, vol. 7, no. 4, pp. 2463–2474, Nov. 2021, doi:](http://paperpile.com/b/I9CICW/0GxH) [10.1007/s40808-020-01005-3.](http://dx.doi.org/10.1007/s40808-020-01005-3.)

[118] [R. George, H. Padalia, S. K. Sinha, and A. S. Kumar, “Evaluating sensitivity of hyperspectral indices for estimating mangrove chlorophyll in Middle Andaman Island, India,” *Environ. Monit. Assess.*, vol. 191, no. Suppl 3, p. 785, Jan. 2020, doi:](http://paperpile.com/b/I9CICW/k53M) [10.1007/s10661-019-7679-6.](http://dx.doi.org/10.1007/s10661-019-7679-6.)

[119] [J. P. Hati *et al.*, “Estimation of vegetation stress in the mangrove forest using AVIRIS-NG airborne hyperspectral data,” *Modeling Earth Systems and Environment*, vol. 7, no. 3, pp. 1877–1889, Sep. 2021, doi:](http://paperpile.com/b/I9CICW/2nUZ) [10.1007/s40808-020-00916-5.](http://dx.doi.org/10.1007/s40808-020-00916-5.)

[120] [L. Wu, L. Wang, C. Shi, and D. Yin, “Detecting mangrove photosynthesis with solar-induced chlorophyll fluorescence,” *Int. J. Remote Sens.*, vol. 43, no. 3, pp. 1037–1053, Feb. 2022, doi:](http://paperpile.com/b/I9CICW/8PhL) [10.1080/01431161.2022.2032457.](http://dx.doi.org/10.1080/01431161.2022.2032457.)

[121] [A. M. Ali, R. Darvishzadeh, K. R. Shahi, and A. Skidmore, “Validating the Predictive Power of Statistical Models in Retrieving Leaf Dry Matter Content of a Coastal Wetland from a Sentinel-2 Image,” *Remote Sensing*, vol. 11, no. 16, p. 1936, Aug. 2019, doi:](http://paperpile.com/b/I9CICW/Wxv9) [10.3390/rs11161936.](http://dx.doi.org/10.3390/rs11161936.)

[122] [J. L. O’Connell, K. B. Byrd, and M. Kelly, “Remotely-sensed indicators of N-related biomass allocation in Schoenoplectus acutus,” *PLoS One*, vol. 9, no. 3, p. e90870, Mar. 2014, doi:](http://paperpile.com/b/I9CICW/8qZ9) [10.1371/journal.pone.0090870.](http://dx.doi.org/10.1371/journal.pone.0090870.)

[123] [X. Kang *et al.*, “Modeling Gross Primary Production of a Typical Coastal Wetland in China Using MODIS Time Series and CO2 Eddy Flux Tower Data,” *Remote Sensing*, vol. 10, no. 5, p. 708, May 2018, doi:](http://paperpile.com/b/I9CICW/fUdN) [10.3390/rs10050708.](http://dx.doi.org/10.3390/rs10050708.)

[124] [N. Lele *et al.*, “Seasonal variation in photosynthetic rates and satellite-based GPP estimation over mangrove forest,” *Environ. Monit. Assess.*, vol. 193, no. 2, p. 61, Jan. 2021, doi:](http://paperpile.com/b/I9CICW/lfXr) [10.1007/s10661-021-08846-0.](http://dx.doi.org/10.1007/s10661-021-08846-0.)

[125] [J. Randall, C. R. Johnson, J. Ross, and J.-P. Hermand, “Acoustic investigation of the primary production of an Australian temperate macroalgal (Ecklonia radiata) system,” *J. Exp. Mar. Bio. Ecol.*, vol. 524, p. 151309, Mar. 2020, doi:](http://paperpile.com/b/I9CICW/IMQ4) [10.1016/j.jembe.2019.151309.](http://dx.doi.org/10.1016/j.jembe.2019.151309.)

[126] [C. J. Wilson, P. S. Wilson, and K. H. Dunton, “An acoustic investigation of seagrass photosynthesis,” *Mar. Biol.*, vol. 159, no. 10, pp. 2311–2322, Oct. 2012, doi:](http://paperpile.com/b/I9CICW/MBWS) [10.1007/s00227-012-2016-4.](http://dx.doi.org/10.1007/s00227-012-2016-4.)

[127] [M. S. Ballard *et al.*, “Application of acoustical remote sensing techniques for ecosystem monitoring of a seagrass meadow,” *J. Acoust. Soc. Am.*, vol. 147, no. 3, p. 2002, Mar. 2020, doi:](http://paperpile.com/b/I9CICW/3Kds) [10.1121/10.0000954.](http://dx.doi.org/10.1121/10.0000954.)

[128] [S. Ghosh and D. R. Mishra, “Analyzing the Long-Term Phenological Trends of Salt Marsh Ecosystem across Coastal LOUISIANA,” *Remote Sensing*, vol. 9, no. 12, p. 1340, Dec. 2017, doi:](http://paperpile.com/b/I9CICW/Gkml) [10.3390/rs9121340.](http://dx.doi.org/10.3390/rs9121340.)

[129] [V. Songsom, W. Koedsin, R. J. Ritchie, and A. Huete, “Mangrove Phenology and Environmental Drivers Derived from Remote Sensing in Southern Thailand,” *Remote Sensing*, vol. 11, no. 8, p. 955, Apr. 2019, doi:](http://paperpile.com/b/I9CICW/ykx2) [10.3390/rs11080955.](http://dx.doi.org/10.3390/rs11080955.)

[130] [A. Fernandez-Carrillo, E. Sanchez-Rodriguez, and V. F. Rodriguez-Galiano, “Characterising marshland temporal dynamics using remote sensing: The case of Bolboschoenetum maritimi in Doñana national park,” *Appl. Geogr.*, vol. 112, p. 102094, Nov. 2019, doi:](http://paperpile.com/b/I9CICW/hmOD) [10.1016/j.apgeog.2019.102094.](http://dx.doi.org/10.1016/j.apgeog.2019.102094.)

[131] [N. Younes, T. D. Northfield, K. E. Joyce, S. W. Maier, N. C. Duke, and L. Lymburner, “A Novel Approach to Modelling Mangrove Phenology from Satellite Images: A Case Study from Northern Australia,” *Remote Sensing*, vol. 12, no. 24, p. 4008, Dec. 2020, doi:](http://paperpile.com/b/I9CICW/WoKe) [10.3390/rs12244008.](http://dx.doi.org/10.3390/rs12244008.)

[132] [D. A. Chamberlain, S. R. Phinn, and H. P. Possingham, “Mangrove Forest Cover and Phenology with Landsat Dense Time Series in Central Queensland, Australia,” *Remote Sensing*, vol. 13, no. 15, p. 3032, Aug. 2021, doi:](http://paperpile.com/b/I9CICW/g3MN) [10.3390/rs13153032.](http://dx.doi.org/10.3390/rs13153032.)

[133] [C. Petus *et al.*, “Estimating the exposure of coral reefs and seagrass meadows to land-sourced contaminants in river flood plumes of the great barrier reef: Validating a simple satellite risk framework with environmental data,” *Remote Sens. (Basel)*, vol. 8, no. 3, p. 210, Mar. 2016, doi:](http://paperpile.com/b/I9CICW/ntOK) [10.3390/rs8030210.](http://dx.doi.org/10.3390/rs8030210.)

[134] [Y. Mo, M. S. Kearney, and J. C. A. Riter, “Post-Deepwater Horizon Oil Spill Monitoring of Louisiana Salt Marshes Using Landsat Imagery,” *Remote Sensing*, vol. 9, no. 6, p. 547, Jun. 2017, doi:](http://paperpile.com/b/I9CICW/f7iU) [10.3390/rs9060547.](http://dx.doi.org/10.3390/rs9060547.)

[135] [B. Adamu, K. Tansey, and B. Ogutu, “Remote sensing for detection and monitoring of vegetation affected by oil spills,” *Int. J. Remote Sens.*, vol. 39, no. 11, pp. 3628–3645, Jun. 2018, doi:](http://paperpile.com/b/I9CICW/zNyZ) [10.1080/01431161.2018.1448483.](http://dx.doi.org/10.1080/01431161.2018.1448483.)

[136] [W. J. Kenworthy, N. Cosentino-Manning, L. Handley, M. Wild, and S. Rouhani, “Seagrass response following exposure to Deepwater Horizon oil in the Chandeleur Islands, Louisiana (USA),” *Mar. Ecol. Prog. Ser.*, vol. 576, pp. 145–161, Aug. 2017, doi:](http://paperpile.com/b/I9CICW/e2GD) [10.3354/meps11983.](http://dx.doi.org/10.3354/meps11983.)

[137] [L. C. M. Santos, M. Cunha-Lignon, Y. Schaeffer-Novelli, and G. Cintrón-Molero, “Long-term effects of oil pollution in mangrove forests (Baixada Santista, Southeast Brazil) detected using a GIS-based multitemporal analysis of aerial photographs,” *Brazil. J. Oceanogr.*, vol. 60, no. 2, pp. 159–170, Jun. 2012, Accessed: Oct. 17, 2022. [Online]. Available:](http://paperpile.com/b/I9CICW/8o6n) <https://www.scielo.br/j/bjoce/a/P7gh7d3wjSZ5LsGtFtsxR5v/abstract/?lang=en>

[138] [S. Khanna, M. J. Santos, S. L. Ustin, A. Koltunov, R. F. Kokaly, and D. A. Roberts, “Detection of salt marsh vegetation stress and recovery after the Deepwater Horizon Oil Spill in Barataria Bay, Gulf of Mexico using AVIRIS data,” *PLoS One*, vol. 8, no. 11, p. e78989, Nov. 2013, doi:](http://paperpile.com/b/I9CICW/Ognt) [10.1371/journal.pone.0078989.](http://dx.doi.org/10.1371/journal.pone.0078989.)

[139] [S. H. Peterson, D. A. Roberts, M. Beland, R. F. Kokaly, and S. L. Ustin, “Oil detection in the coastal marshes of Louisiana using MESMA applied to band subsets of AVIRIS data,” *Remote Sens. Environ.*, vol. 159, pp. 222–231, Mar. 2015, doi:](http://paperpile.com/b/I9CICW/LCfe) [10.1016/j.rse.2014.12.009.](http://dx.doi.org/10.1016/j.rse.2014.12.009.)

[140] [J. L. O’Connell, D. R. Mishra, D. L. Cotten, L. Wang, and M. Alber, “The Tidal Marsh Inundation Index (TMII): An inundation filter to flag flooded pixels and improve MODIS tidal marsh vegetation time-series analysis,” *Remote Sens. Environ.*, vol. 201, no. July, pp. 34–46, Nov. 2017, doi:](http://paperpile.com/b/I9CICW/M4Sh) [10.1016/j.rse.2017.08.008.](http://dx.doi.org/10.1016/j.rse.2017.08.008.)

[141] [E. Asbridge and R. M. Lucas, “Mangrove Response to Environmental Change in Kakadu National Park,” *IEEE Journal of Selected Topics in Applied Earth Observations and Remote Sensing*, vol. 9, no. 12, pp. 5612–5620, Dec. 2016, doi:](http://paperpile.com/b/I9CICW/b5sg) [10.1109/JSTARS.2016.2616449.](http://dx.doi.org/10.1109/JSTARS.2016.2616449.)

[142] [D. P. Ward *et al.*, “Floodplain inundation and vegetation dynamics in the Alligator Rivers region (Kakadu) of northern Australia assessed using optical and radar remote sensing,” *Remote Sens. Environ.*, vol. 147, pp. 43–55, May 2014, doi:](http://paperpile.com/b/I9CICW/9Lp3) [10.1016/j.rse.2014.02.009.](http://dx.doi.org/10.1016/j.rse.2014.02.009.)

[143] [R. F. Thomas, R. T. Kingsford, Y. Lu, S. J. Cox, N. C. Sims, and S. J. Hunter, “Mapping inundation in the heterogeneous floodplain wetlands of the Macquarie Marshes, using Landsat Thematic Mapper,” *J. Hydrol.*, vol. 524, pp. 194–213, May 2015, doi:](http://paperpile.com/b/I9CICW/QFcc) [10.1016/j.jhydrol.2015.02.029.](http://dx.doi.org/10.1016/j.jhydrol.2015.02.029.)

[144] [D. C. Williams and J. G. Lyon, “Historical aerial photographs and a geographic information system (GIS) to determine effects of long-term water level fluctuations on wetlands along the St. Marys River, Michigan, USA,” *Aquat. Bot.*, vol. 58, no. 3, pp. 363–378, Oct. 1997, doi:](http://paperpile.com/b/I9CICW/zabG) [10.1016/S0304-3770(97)00046-6.](http://dx.doi.org/10.1016/S0304-3770(97)00046-6.)

[145] [P. L. A. Erftemeijer and O. Hamerlynck, “Die-Back of the Mangrove Heritiera littoralis Dryand, in the Rufiji Delta (Tanzania) Following El Niño Floods,” *J. Coast. Res.*, pp. 228–235, 2005, [Online]. Available:](http://paperpile.com/b/I9CICW/VPeA) <http://www.jstor.org/stable/25736988>

[146] [M. S. H. Mandal and T. Hosaka, “Assessing cyclone disturbances (1988–2016) in the Sundarbans mangrove forests using Landsat and Google Earth Engine,” *Nat. Hazards*, vol. 102, no. 1, pp. 133–150, May 2020, doi:](http://paperpile.com/b/I9CICW/lPCC) [10.1007/s11069-020-03914-z.](http://dx.doi.org/10.1007/s11069-020-03914-z.)

[147] [M. J. C. Buitre, H. Zhang, and H. Lin, “The Mangrove Forests Change and Impacts from Tropical Cyclones in the Philippines Using Time Series Satellite Imagery,” *Remote Sensing*, vol. 11, no. 6, p. 688, Mar. 2019, doi:](http://paperpile.com/b/I9CICW/BZLE) [10.3390/rs11060688.](http://dx.doi.org/10.3390/rs11060688.)

[148] [H. Roemer, G. Kaiser, H. Sterr, and R. Ludwig, “Using remote sensing to assess tsunami-induced impacts on coastal forest ecosystems at the Andaman Sea coast of Thailand,” *Nat. Hazards Earth Syst. Sci.*, vol. 10, no. 4, pp. 729–745, Apr. 2010, doi:](http://paperpile.com/b/I9CICW/RjlR) [10.5194/nhess-10-729-2010.](http://dx.doi.org/10.5194/nhess-10-729-2010.)

[149] [J. P. Serrano-Rubio, M. D. M. Ruiz, and U. Vidal-Espitia, “Integrating remote sensing and image processing to test for disturbance effects in a post-hurricane mangrove ecosystem,” *J. VLSI Signal Process. Syst. Signal Image Video Technol.*, vol. 15, no. 2, pp. 351–359, Mar. 2021, doi:](http://paperpile.com/b/I9CICW/CEXs) [10.1007/s11760-020-01754-9.](http://dx.doi.org/10.1007/s11760-020-01754-9.)

[150] [E. Hernández, E. Cuevas, S. Pinto-Pacheco, and G. Ortíz-Ramírez, “You can bend me but can’t break me: Vegetation regeneration after hurricane María passed over an urban coastal wetland in northeastern Puerto Rico,” *Front. For. Glob. Chang.*, vol. 4, Nov. 2021, doi:](http://paperpile.com/b/I9CICW/aKv7) [10.3389/ffgc.2021.752328.](http://dx.doi.org/10.3389/ffgc.2021.752328.)

[151] [M. C. L. Cohen *et al.*, “Effects of the 2017–2018 winter freeze on the northern limit of the American mangroves, Mississippi River delta plain,” *Geomorphology* , vol. 394, p. 107968, Dec. 2021, doi:](http://paperpile.com/b/I9CICW/y0lg) [10.1016/j.geomorph.2021.107968.](http://dx.doi.org/10.1016/j.geomorph.2021.107968.)

[152] [N. N. Salghuna and R. C. P. Pillutla, “Mapping Mangrove Species Using Hyperspectral Data: A Case Study of Pichavaram Mangrove Ecosystem, Tamil Nadu,” *Earth Systems and Environment*, vol. 1, no. 2, p. 24, Nov. 2017, doi:](http://paperpile.com/b/I9CICW/DXwQ) [10.1007/s41748-017-0024-8.](http://dx.doi.org/10.1007/s41748-017-0024-8.)

[153] [D. Rice, J. Rooth, and J. C. Stevenson, “Colonization and expansion of Phragmites australis in upper Chesapeake Bay tidal marshes,” *Wetlands*, vol. 20, no. 2, pp. 280–299, Jun. 2000, doi:](http://paperpile.com/b/I9CICW/a5Yk) [10.1672/0277-5212(2000)020[0280:CAEOPA]2.0.CO;2.](http://dx.doi.org/10.1672/0277-5212(2000)020%5B0280:CAEOPA%5D2.0.CO;2.)

[154] [T. Abeysinghe *et al.*, “Mapping Invasive Phragmites australis in the Old Woman Creek Estuary Using UAV Remote Sensing and Machine Learning Classifiers,” *Remote Sensing*, vol. 11, no. 11, p. 1380, Jun. 2019, doi:](http://paperpile.com/b/I9CICW/ohzo) [10.3390/rs11111380.](http://dx.doi.org/10.3390/rs11111380.)

[155] [M. R. Nepita-Villanueva, C. A. Berlanga-Robles, A. Ruiz-Luna, and J. H. Morales Barcenas, “Spatio-temporal mangrove canopy variation (2001–2016) assessed using the MODIS enhanced vegetation index (EVI),” *J. Coast. Conserv.*, vol. 23, no. 3, pp. 589–597, Jun. 2019, doi:](http://paperpile.com/b/I9CICW/TK1R) [10.1007/s11852-019-00689-9.](http://dx.doi.org/10.1007/s11852-019-00689-9.)

[156] [E. I. Paling, H. T. Kobryn, and G. Humphreys, “Assessing the extent of mangrove change caused by Cyclone Vance in the eastern Exmouth Gulf, northwestern Australia,” *Estuar. Coast. Shelf Sci.*, vol. 77, no. 4, pp. 603–613, May 2008, doi:](http://paperpile.com/b/I9CICW/izbX) [10.1016/j.ecss.2007.10.019.](http://dx.doi.org/10.1016/j.ecss.2007.10.019.)

[157] [M. B. Fernandes *et al.*, “Landsat historical records reveal large-scale dynamics and enduring recovery of seagrasses in an impacted seascape,” *Sci. Total Environ.*, vol. 813, p. 152646, Mar. 2022, doi:](http://paperpile.com/b/I9CICW/vedF) [10.1016/j.scitotenv.2021.152646.](http://dx.doi.org/10.1016/j.scitotenv.2021.152646.)

[158] [M. Jia, Z. Wang, D. Liu, C. Ren, X. Tang, and Z. Dong, “Monitoring loss and recovery of salt marshes in the Liao river delta, China,” *J. Coast. Res.*, vol. 300, no. 2, pp. 371–377, Mar. 2015, doi:](http://paperpile.com/b/I9CICW/8N7d) [10.2112/jcoastres-d-13-00056.1.](http://dx.doi.org/10.2112/jcoastres-d-13-00056.1.)

[159] [H. Römer, J. Jeewarongkakul, G. Kaiser, R. Ludwig, and H. Sterr, “Monitoring post-tsunami vegetation recovery in Phang-Nga province, Thailand, based on IKONOS imagery and field investigations – a contribution to the analysis of tsunami vulnerability of coastal ecosystems,” *Int. J. Remote Sens.*, vol. 33, no. 10, pp. 3090–3121, May 2012, doi:](http://paperpile.com/b/I9CICW/7rCo) [10.1080/01431161.2011.628710.](http://dx.doi.org/10.1080/01431161.2011.628710.)

[160] [B. Proença *et al.*, “Potential of High-Resolution Pléiades Imagery to Monitor Salt Marsh Evolution After Spartina Invasion,” *Remote Sensing*, vol. 11, no. 8, p. 968, Apr. 2019, doi:](http://paperpile.com/b/I9CICW/3zpY) [10.3390/rs11080968.](http://dx.doi.org/10.3390/rs11080968.)

[161] [A. J. Meehan and R. J. West, “Recovery times for a damaged Posidonia australis bed in south eastern Australia,” *Aquat. Bot.*, vol. 67, no. 2, pp. 161–167, Jun. 2000, doi:](http://paperpile.com/b/I9CICW/WKNq) [10.1016/S0304-3770(99)00097-2.](http://dx.doi.org/10.1016/S0304-3770(99)00097-2.)

[162] [M. Jeanson, E. J. Anthony, F. Dolique, and C. Cremades, “Mangrove Evolution in Mayotte Island, Indian Ocean: A 60-year Synopsis Based on Aerial Photographs,” *Wetlands*, vol. 34, no. 3, pp. 459–468, Jun. 2014, doi:](http://paperpile.com/b/I9CICW/F4eh) [10.1007/s13157-014-0512-7.](http://dx.doi.org/10.1007/s13157-014-0512-7.)

[163] [S. Khanna, M. J. Santos, A. Koltunov, K. D. Shapiro, M. Lay, and S. L. Ustin, “Marsh Loss Due to Cumulative Impacts of Hurricane Isaac and the Deepwater Horizon Oil Spill in Louisiana,” *Remote Sensing*, vol. 9, no. 2, p. 169, Feb. 2017, doi:](http://paperpile.com/b/I9CICW/eCG3) [10.3390/rs9020169.](http://dx.doi.org/10.3390/rs9020169.)

[164] [K. Shapiro, S. Khanna, and S. L. Ustin, “Vegetation Impact and Recovery from Oil-Induced Stress on Three Ecologically Distinct Wetland Sites in the Gulf of Mexico,” *J. Mar. Sci. Eng.*, vol. 4, no. 2, p. 33, May 2016, doi:](http://paperpile.com/b/I9CICW/Rgd9) [10.3390/jmse4020033.](http://dx.doi.org/10.3390/jmse4020033.)

[165] [W. Nardin *et al.*, “Seasonality and Characterization Mapping of Restored Tidal Marsh by NDVI Imageries Coupling UAVs and Multispectral Camera,” *Remote Sensing*, vol. 13, no. 21, p. 4207, Oct. 2021, doi:](http://paperpile.com/b/I9CICW/bQRu) [10.3390/rs13214207.](http://dx.doi.org/10.3390/rs13214207.)

[166] [L. Meng, S. Zhou, H. Zhang, and X. Bi, “Estimating soil salinity in different landscapes of the Yellow River Delta through Landsat OLI/TIRS and ETM+ Data,” *J. Coast. Conserv.*, vol. 20, no. 4, pp. 271–279, Aug. 2016, doi:](http://paperpile.com/b/I9CICW/oFou) [10.1007/s11852-016-0437-9.](http://dx.doi.org/10.1007/s11852-016-0437-9.)

[167] [T. D. Pham *et al.*, “Improvement of Mangrove Soil Carbon Stocks Estimation in North Vietnam Using Sentinel-2 Data and Machine Learning Approach,” *GISci. Remote Sens.*, vol. 58, no. 1, pp. 68–87, Jan. 2021, doi:](http://paperpile.com/b/I9CICW/jm28) [10.1080/15481603.2020.1857623.](http://dx.doi.org/10.1080/15481603.2020.1857623.)

[168] [I. Salehi Hikouei, S. S. Kim, and D. R. Mishra, “Machine-Learning Classification of Soil Bulk Density in Salt Marsh Environments,” *Sensors* , vol. 21, no. 13, Jun. 2021, doi:](http://paperpile.com/b/I9CICW/vxCS) [10.3390/s21134408.](http://dx.doi.org/10.3390/s21134408.)

[169] [N. J. P. Anne, A. H. Abd-Elrahman, D. B. Lewis, and N. A. Hewitt, “Modeling soil parameters using hyperspectral image reflectance in subtropical coastal wetlands,” *Int. J. Appl. Earth Obs. Geoinf.*, vol. 33, pp. 47–56, Dec. 2014, doi:](http://paperpile.com/b/I9CICW/3Zt0) [10.1016/j.jag.2014.04.007.](http://dx.doi.org/10.1016/j.jag.2014.04.007.)

[170] [R.-M. Yang and W.-W. Guo, “Using time-series Sentinel-1 data for soil prediction on invaded coastal wetlands,” *Environ. Monit. Assess.*, vol. 191, no. 7, p. 462, Jun. 2019, doi:](http://paperpile.com/b/I9CICW/Ot6t) [10.1007/s10661-019-7580-3.](http://dx.doi.org/10.1007/s10661-019-7580-3.)

[171] [R.-M. Yang, W.-W. Guo, and J.-B. Zheng, “Soil prediction for coastal wetlands following Spartina alterniflora invasion using Sentinel-1 imagery and structural equation modeling,” *Catena*, vol. 173, pp. 465–470, Feb. 2019, doi:](http://paperpile.com/b/I9CICW/RmHn) [10.1016/j.catena.2018.10.045.](http://dx.doi.org/10.1016/j.catena.2018.10.045.)

[172] [R.-M. Yang and W.-W. Guo, “Modelling of soil organic carbon and bulk density in invaded coastal wetlands using Sentinel-1 imagery,” *Int. J. Appl. Earth Obs. Geoinf.*, vol. 82, p. 101906, Oct. 2019, doi:](http://paperpile.com/b/I9CICW/K8OW) [10.1016/j.jag.2019.101906.](http://dx.doi.org/10.1016/j.jag.2019.101906.)

[173] [J. M. Knight, P. E. R. Dale, J. Spencer, and L. Griffin, “Exploring LiDAR data for mapping the micro-topography and tidal hydro-dynamics of mangrove systems: An example from southeast Queensland, Australia,” *Estuar. Coast. Shelf Sci.*, vol. 85, no. 4, pp. 593–600, Dec. 2009, doi:](http://paperpile.com/b/I9CICW/fF14) [10.1016/j.ecss.2009.10.002.](http://dx.doi.org/10.1016/j.ecss.2009.10.002.)

[174] [M. Young, D. Ierodiaconou, and T. Womersley, “Forests of the sea: Predictive habitat modelling to assess the abundance of canopy forming kelp forests on temperate reefs,” *Remote Sens. Environ.*, vol. 170, pp. 178–187, Dec. 2015, doi:](http://paperpile.com/b/I9CICW/8UDL) [10.1016/j.rse.2015.09.020.](http://dx.doi.org/10.1016/j.rse.2015.09.020.)

[175] [J. N. Rogers, C. E. Parrish, L. G. Ward, and D. M. Burdick, “Improving salt marsh digital elevation model accuracy with full-waveform lidar and nonparametric predictive modeling,” *Estuar. Coast. Shelf Sci.*, vol. 202, pp. 193–211, Mar. 2018, doi:](http://paperpile.com/b/I9CICW/mZCS) [10.1016/j.ecss.2017.11.034.](http://dx.doi.org/10.1016/j.ecss.2017.11.034.)

[176] [B. R. Couvillion and H. Beck, “Marsh collapse thresholds for coastal Louisiana estimated using elevation and vegetation index data,” *J. Coast. Res.*, vol. 63, pp. 58–67, Apr. 2013, doi:](http://paperpile.com/b/I9CICW/cqDz) [10.2112/si63-006.1.](http://dx.doi.org/10.2112/si63-006.1.)
